# Escalating morphine dosing in HIV-1 Tat transgenic mice with sustained Tat exposure reveals an allostatic shift in neuroinflammatory regulation accompanied by increased neuroprotective non-endocannabinoid lipid signaling molecules and amino acids

**DOI:** 10.1186/s12974-020-01971-6

**Published:** 2020-11-18

**Authors:** Douglas J. Hermes, Ian R. Jacobs, Megan C. Key, Alexis F. League, Barkha J. Yadav-Samudrala, Changqing Xu, Virginia D. McLane, Sara R. Nass, Wei Jiang, Rick B. Meeker, Bogna M. Ignatowska-Jankowska, Aron H. Lichtman, Zibo Li, Zhanhong Wu, Hong Yuan, Pamela E. Knapp, Kurt F. Hauser, Sylvia Fitting

**Affiliations:** 1 Department of Psychology & Neuroscience, University of North Carolina, Chapel Hill, NC USA; 2 Department of Pharmacology & Toxicology, Virginia Commonwealth University, Richmond, VA USA; 3 Department of Microbiology and Immunology, Medical University of South Carolina, Charleston, SC USA; 4 Division of Infectious Diseases, Department of Medicine, Medical University of South Carolina, Charleston, SC USA; 5 Department of Neurology, University of North Carolina, Chapel Hill, NC USA; 6 Okinawa Institute of Science and Technology, Neuronal Rhythms in Movement Unit, Okinawa, 904-0495 Japan; 7 Department of Radiology, School of Medicine, University of North Carolina, Chapel Hill, NC USA; 8 Department of Anatomy & Neurobiology, Virginia Commonwealth University, Richmond, VA USA

**Keywords:** Opioid drug abuse, [^18^F]-PBR111 PET imaging, Endocannabinoids, Peroxisome proliferator activator receptor α (PPAR-α) agonists, Microgliosis, Cytokines, Chemokines, Anti-inflammation, Proinflammation, Phenylalanine, Aliphatic side-chain amino acids

## Abstract

**Background:**

Human immunodeficiency virus type-1 (HIV-1) and opiates cause long-term inflammatory insult to the central nervous system (CNS) and worsen disease progression and HIV-1-related neuropathology. The combination of these proinflammatory factors reflects a devastating problem as opioids have high abuse liability and continue to be prescribed for certain patients experiencing HIV-1-related pain.

**Methods:**

Here, we examined the impact of chronic (3-month) HIV-1 transactivator of transcription (Tat) exposure to short-term (8-day), escalating morphine in HIV-1 Tat transgenic mice that express the HIV-1 Tat protein in a GFAP promoter-regulated, doxycycline (DOX)-inducible manner. In addition to assessing morphine-induced tolerance in nociceptive responses organized at spinal (i.e., tail-flick) and supraspinal (i.e., hot-plate) levels, we evaluated neuroinflammation via positron emission tomography (PET) imaging using the [^18^F]-PBR111 ligand, immunohistochemistry, and cytokine analyses. Further, we examined endocannabinoid (eCB) levels, related non-eCB lipids, and amino acids via mass spectrometry.

**Results:**

Tat-expressing [Tat(+)] transgenic mice displayed antinociceptive tolerance in the tail withdrawal and hot-plate assays compared to control mice lacking Tat [Tat(−)]. This tolerance was accompanied by morphine-dependent increases in Iba-1 ± 3-nitrotryosine immunoreactive microglia, and alterations in pro- and anti-inflammatory cytokines, and chemokines in the spinal cord and striatum, while increases in neuroinflammation were absent by PET imaging of [^18^F]-PBR111 uptake. Tat and morphine exposure differentially affected eCB levels, non-eCB lipids, and specific amino acids in a region-dependent manner. In the striatum, non-eCB lipids were significantly increased by short-term, escalating morphine exposure, including peroxisome proliferator activator receptor alpha (PPAR-α) ligands *N*-oleoyl ethanolamide (OEA) and *N*-palmitoyl ethanolamide (PEA), as well as the amino acids phenylalanine and proline. In the spinal cord, Tat exposure increased amino acids leucine and valine, while morphine decreased levels of tyrosine and valine but did not affect eCBs or non-eCB lipids.

**Conclusion:**

Overall results demonstrate that 3 months of Tat exposure increased morphine tolerance and potentially innate immune tolerance evidenced by reductions in specific cytokines (e.g., IL-1α, IL-12p40) and microglial reactivity. In contrast, short-term, escalating morphine exposure acted as a secondary stressor revealing an allostatic shift in CNS baseline inflammatory responsiveness from sustained Tat exposure.

## Background

Studies investigating the effects of opiate abuse, including prescription drugs, in human immunodeficiency virus type-1 (HIV-1)–infected patients demonstrate multiple comorbid interactions of opiates on the HIV-1-infected nervous system. Opiates promote HIV-1-related disease progression [1–4], HIV-1-associated neuropathy [5–8], and HIV-1 encephalopathy, especially prior to the introduction of combined antiretroviral therapy (pre-cART) [9–14]. The deleterious effects of opiates on neuroHIV in the central nervous system (CNS) include increased inflammatory products within infected glia that contribute to bystander dysfunction and toxicity in uninfected neurons and glia (for detailed reviews, please see [7, 13, 15, 16]). Since the inception of cART, the impact of chronic inflammatory insult by HIV-1 infection has become an important topic; persistent latently and productively infected perivascular macrophage and microglial populations [14, 17–19] represent the primary reservoirs for HIV-1 in the CNS [20–24]. In vivo and in vitro studies focusing on the HIV-1 transactivator of transcription (Tat) protein demonstrate morphine’s exacerbating effects on microglial activation [25–29], astroglia dysregulation [29–32], cytokine production [33–38], and blood-brain barrier (BBB) breakdown [13, 39, 40], with additional effects on oxidative stress [33, 34, 41, 42], intracellular calcium [34, 37, 43], and neurotoxicity [38, 42–44], potentially due to morphine’s action on μ-opioid receptor (MOR)-expressing glia [31, 45]. Notably, HIV-1 and HIV-1 proteins, such as Tat, impact opioid gene expression and splicing specificity [38, 46–48], potentially mediated through the release of various proinflammatory cytokines, including IL-6, TNF, GM-CSF, and IFN-γ [49, 50]. Further, the proinflammatory effects of HIV-1 Tat at C-C chemokine receptor type 5 (CCR5) desensitize MOR or δ-opioid receptors (DOR [51–53]), an effect that appears to contribute to decreased antinociceptive potency of morphine in Tat transgenic mice [54]. Interestingly, recent research has demonstrated long-term immune tolerance to repeated inflammatory insult within resident microglia in the CNS, which could impact the response to morphine after chronic Tat exposure [55–58].

Additionally, changes in various small molecules, such as endocannabinoids (eCBs) as well as non-eCB lipid signaling molecules and amino acids have been implicated in opioid-induced antinociception, tolerance, and dependence [59–66]. Morphine exposure leads to upregulated cannabinoid receptor type 1 (CB_1_R) expression [62, 67, 68], while CB_1_R knockout mice display markedly reduced reinforcing/rewarding responses to morphine or heroin, and inhibitors of eCB degradative enzymes reduce opioid withdrawal as well as opioid-seeking behavior in mice [61, 62, 64]. Morphine-tolerant rodents show complex changes in eCB levels and/or related non-eCB lipid signaling molecules (e.g., *N*-oleoyl ethanolamide; OEA and *N*-palmitoyl ethanolamide; PEA), with reports ranging from upregulation to downregulation depending on the brain region, duration of exposure, and the particular eCB(s) or lipid(s) assessed [69–72]. Similarly, the levels of specific amino acids have been demonstrated to be altered in the brains of rodents exposed to opioids [59, 60]. When given as dietary supplements, tyrosine, l-glutamine, and l-5-hydroxytryptophan allay some of the physical and emotional aspects of opioid withdrawal, presumably by restoring neurotransmitter levels to normative values [73]. Some amino acids (e.g., proline and phenylalanine) are known for their anti-inflammatory and analgesic properties [74, 75], although high plasma concentrations of branched-chain amino acids (BCAA) above 2 mmol/L can contribute to proinflammatory effects and oxidative stress [76]. Further, eCBs and related non-eCB lipids possess neuroprotective, anti-inflammatory, and neurotrophic properties that likely play a role in various neurodegenerative diseases, including Parkinson’s and Alzheimer’s disease models [77–81]. However, the interactive effects of chronic HIV-1 Tat and morphine exposure on pathophysiological changes in eCBs and related non-eCB lipids have not been investigated in detail.

In the present study, we assessed morphine tolerance in the spinal cord-mediated tail-flick task and a supraspinal hot-plate assay in the Tat transgenic mouse model with chronic induction of Tat expression for 3 months and administration of a short-term 8-day escalating morphine regimen. To examine neuroinflammation, positron emission tomography (PET) imaging experiments were conducted using the 2-(6-chloro-2-(4-(3-^18^F-fluoropropoxy)phenyl)imidazo[1,2-a]pyri-din-3-yl)-*N*,*N*-diethylacetamide ([^18^F]-PRB111) imaging probe that specifically binds to the peripheral benzodiazepine receptor (PBR), recently named as the 18-kDa translocator protein (TSPO). [^18^F]-PRB111 is a second-generation PET ligand for TSPO and a promising imaging agent for TSPO expression in neurodegenerative disorders [82–84]. Additionally, we identified activated microglia using ionized calcium binding adaptor molecule 1 (Iba-1) and 3-nitrotyrosine (3-NT) immunoreactivity, and measured cytokines by multiplex immunoassay in the striatum and spinal cord of Tat transgenic mice. To determine the impact of HIV-1 Tat and opioid exposure on the eCB system in these two CNS regions, we examined 2-arachidonoyl glycerol (2-AG), *N*-arachidonoyl ethanolamide (AEA), and related non-eCB lipids in Tat transgenic mice via mass spectrometry. Lastly, mass spectrometry analyses assessed alterations in the concentration of multiple amino acids in the spinal cord and striatum of Tat transgenic mice. We hypothesized that HIV-1 Tat expression attenuates morphine-induced antinociceptive tolerance after morphine exposure, potentially due to alterations of inflammatory processes and changes in expression levels of eCBs, related non-eCB lipids, and amino acids.

## Materials and methods

### Animals

Doxycycline (DOX)-inducible, brain-specific HIV-1_IIIB_ Tat_1-86_ transgenic mice (~ 3 months of age, ~ 25 g, males) were used in the present study and developed on a C57BL/6J hybrid background as previously described [29, 85]. To induce Tat expression in mice that express the *GFAP-rtTA* and *TRE-tat* genes [Tat(+) mice], animals were fed a specially formulated chow containing 6 mg/g DOX (Harlan, Indianapolis, IN, product #: TD.09282). Control Tat(−) transgenic mice that express only the *GFAP-rtTA* gene and lack the *tat* transgene received the same DOX diet. Mice were fed the DOX-supplemented food for up to 3 months before experiments were conducted. Mice had free access to water and chow and were group-housed (2–4 mice per cage) on a reversed 12 h light/dark cycle (lights off at 8:00 AM). All animal procedures were approved by the University of North Carolina at Chapel Hill (UNC) Institutional Animal Care and Use Committee (IACUC) and were in keeping with ethical guidelines defined by the National Institutes of Health (NIH Publication No. 85-23).

### Repeated Escalating Morphine Injections

Morphine sulfate was obtained from the National Institutes of Health National Institute on Drug Abuse (Drug Supply System, Bethesda, MD) and dissolved in 0.9% physiological saline. Saline and morphine were administered via the subcutaneous (s.c.) route in an injection volume of 10 mL/kg. To induce tolerance, morphine was administered in escalating doses via twice daily injections for 8 days (8:00 AM and 6:00 PM): 10 mg/kg on day 1, 20 mg/kg on days 2 and 3, 40 mg/kg on days 4 and 5, 80 mg/kg on day 6, and 160 mg/kg on days 7 and 8, based on previous studies using similar escalating injection regimens [86, 87]. Saline-treated animals received an equivalent volume of sterile, 0.9% physiological saline.

### Behavioral tolerance studies

#### Acute cumulative morphine injections

**Fig. 1:**
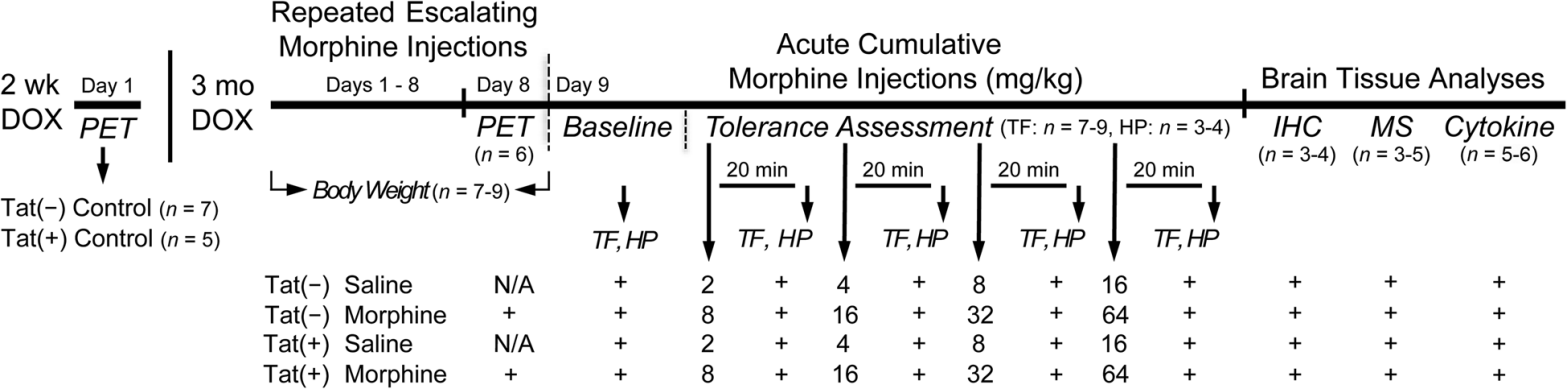
Experimental design depicted on a timeline. All Tat transgenic mice received DOX-containing chow for 3 months and were then subcutaneously (s.c.) injected with saline or morphine for 8 days, except for a separate set of mice that were PET imaged as control animals (no injections), which received DOX for 2 weeks. Body mass was recorded daily. On day 9 after morphine administration, mice were tested for baseline activity in the tail-flick and hot-plate assays. This was followed by four acute, cumulative s.c. morphine injections with a 20-min wait period after each injection before tail-flick responses to warm-water and hindpaw lick or lift to a heated hot-plate were tested. At the end of behavioral testing, animals were sacrificed immediately and brains were taken for immunohistochemistry and mass spectrometry analysis. Cytokine analyses were conducted on a separate set of animals. *TF* tail-flick assay, *HP* hot-plate assay, *PET* positron emission tomography, *MS* mass spectrometry, *Cytokine* cytokine analyses; *n* = mice per group

To assess opioid tolerance, acute cumulative morphine dose-response curves were evaluated in mice that received the 8-day escalating morphine dosing regimen or eight days of saline injections. On test day (i.e., day 9 at 8:00 AM), the saline-injected group received cumulative s.c. morphine doses of 2, 4, 8, and 16 mg/kg, whereas the morphine-exposed group received cumulative morphine doses of 8, 16, 32, and 64 mg/kg (Fig. 1). Mice were tested before and immediately after each acute cumulative s.c. morphine injection. The tail-flick and hot-plate assays were used to test for tolerance to morphine’s antinociceptive effects. Baseline responses for tail-flick and hot-plate activity were measured on test day 9 before animals received acute cumulative s.c. morphine injections. Following a 20-min absorption period after each injection, tail-flick responses were recorded followed by hot-plate responses. After the last injection, mice were sacrificed and the brains were harvested and processed for immunohistochemistry, cytokine analysis, eCB and related non-eCB lipid analysis, and amino acid analysis.

#### Tail-flick and hot-plate tests

The tail-flick test was conducted on Tat transgenic mice (*n* = 7–9 per group) using a water bath with the temperature maintained at 56 ± 0.1 °C. For baseline latency, tail withdrawal was measured on day 9 before acute cumulative morphine injections. The distal 1/3 of the mouse-tail was immersed in the warm-water bath and the latency for the mouse to remove its tail was measured. The duration of time the tail remained in the water bath was counted as the baseline latency. Baseline latency reaction times in mice before acute cumulative morphine injections were 2 to 3 s. Test latency was evaluated 20 min after each cumulative morphine injection with the latency to remove the tail increasing proportionally to morphine’s analgesic potency. A 10-s maximum cut-off latency was used to prevent tissue damage.

Immediately after the tail-flick test, a subset of animals (*n* = 3–4 per group) were evaluated in the hot-plate test of nociception. Subjects were gently placed on the surface of the hot plate (55 ± 0.1 °C; IITC, Inc., MOD 39). Round Plexiglas™ walls (15 cm high, 10 cm diameter) surrounded the hot plate to prevent escape. Latency to lick or lift a hindpaw was the dependent measure. Hot-plate baselines were taken before acute cumulative morphine injections and after tail-flick assessment with baseline latency reaction times ranging from 4 to 8 s. Test latency was obtained after each cumulative morphine injection following the tail-flick assessment. A 20-s maximum cut-off latency was used to prevent tissue damage.

Antinociception in the tail-flick and hot-plate assays were quantified as the percentage of maximum possible effect (%MPE), which was calculated as %MPE = [(test latency − control latency) / (maximum cut-off latency − control latency)^−1^] × 100 [88].

### Positron emission tomography analysis

All animal positron emission tomography (PET) and computed tomography (CT) imaging were conducted in the Small Animal Imaging Facility of the Biomedical Research Imaging Center (BRIC) at UNC.

The synthesis of [^18^F]-PBR111 was performed as previously described [89–91]. The [^18^F]-PBR111 probe was produced at the BRIC Cyclotron and Radiochemistry Core facility according to the well-established radiosynthesis method [83, 84]. Radiochemical purity was > 95% and the specific activity was 219.78 ± 35.89 GBq/μmol.

PET/CT image acquisition was performed using a small animal PET/CT scanner (Argus, Sedecal, Inc. Spain) with a spatial resolution of 1.2 mm in the center field of view. Tat transgenic mice (*n* = 5–7 per group) were anesthetized with inhalation of 1.5–2.5% isoflurane-oxygen gas mixture. The tail vein was catheterized for radiotracer injection. A dose of [^18^F]-PBR111 (~ 11 MBq in 100 μL) was administered through the mouse tail vein. At 40-min post injection, a 20-min PET acquisition was conducted after a CT scan. PET images were reconstructed using the 2D-OSEM algorithms with scatter, attenuation, and decay correction. Standardized uptake value (SUV) was calculated voxel wise by normalizing the signal to the injection dose and animal body mass. After PET/CT images, brains were collected and the distribution of [^18^F]-PBR111 binding within the brain in higher resolution was assessed using autoradiography following standard procedures. Briefly, brain specimens were snap-frozen and cryo-sectioned at 12 μm thickness. The sections were placed on a phosphor screen for overnight exposure, and autoradiography images were obtained after digital scan using a phosphor imaging system (Cyclone Plus, PerkinElmer Inc.). Standard T2-weighted magnetic resonance images were acquired from eight Tat transgenic mice to form an averaged magnetic resonance imaging (MRI) atlas and provide the substructure reference within brains for the PET images.

For PET data analysis, PET images were first registered to the MRI atlas images via co-registered CT images. Regions of dorsal striatum, hippocampus, frontal cortex, and cerebellum were first drawn in the MRI images as the volumes of interest (VOIs) and superimposed to PET images. Uptake level in each brain VOI was expressed as SUV. The mean SUV and variation were calculated for each group.

### Immunohistochemistry: microglia (Iba-1+) and activated, 3-nitrotyrosine microglia (3-NT+ Iba-1+)

Half of the Tat transgenic mice (*n* = 3–4 per group) were anesthetized with isoflurane and perfused with 4% paraformaldehyde. Brains and the spinal cord (including C1 to C5 as well as the lumbar region from L1 to L5) were removed and post-fixed in 4% paraformaldehyde (for 6 h at 4 °C), then washed in 1× phosphate-buffered saline (PBS) several times and incubated for at least 32 h in 20% sucrose in PBS. Brains were embedded in Tissue-Tek O.C.T. compound, frozen, and stored at − 80 °C until cut. Sagittal brain sections (30 μm) containing the frontal cortex, hippocampus, dorsal striatum, and cerebellum, and coronal spinal cord sections (30 μm, portions of the C1–C5, and L1–L5) were cut on a Leica CM3050S cryostat (Leica, Deerfield, IL). Sections were first incubated in 0.5% H_2_O_2_ for 30 min, in 1% H_2_O_2_ for 60 min, and again in 0.5% H_2_O_2_ for 30 min, incubated for 30 min in permeability solution (PBS with 0.2% Triton X-100 and 1% bovine serum albumin; performed for 3-NT staining only), followed by exposure to blocking buffer for 1 h (PBS with 3% normal goat serum and 0.5% Triton X-100 for Iba1 staining, or 1% normal goat serum and 4% bovine serum albumin for 3-NT staining). Sections were then incubated with the primary antibodies against Iba-1 (rabbit, Wako, #019-19741; 1:500) for the detection of microglia and/or 3-NT (mouse, Santa Cruz, #SC-32757; 1:100), a reactive nitrosyl product formed by reactive nitrogen species such as peroxynitrite, diluted in blocking buffer containing normal goat serum, overnight at 4 °C. The primary antibodies were detected using goat-anti-rabbit Alexa 488 (ThermoFisher, #A-11034, 1:500) and/or goat-anti-mouse Alexa 594 (ThermoFisher, #A-11032, 1:500). The secondary antibody was diluted in goat blocking buffer and applied to the sections for 1 h at room temperature. Cell nuclei were visualized with Hoechst 33342 (Molecular Probes, H3570, exposed for 3 min). Tissue sections were washed thoroughly with PBS and coverslipped with antifade mounting medium (VectaShield, #H-1400). Confocal immunofluorescent images were acquired using a Zeiss LSM T-PMT laser scanning confocal microscope (Zeiss, Thornwood, NY) equipped with a × 20 objective. Images were collected using ZEN 2010 Blue Edition software (Carl Zeiss, Inc., Thornwood, NY). For the frontal cortex, hippocampus, striatum, and cerebellum, one image was sampled per section from ≥ 4 sagittal sections, spaced 300 μm apart, per animal. For the coronal spinal cord sections, one image was sampled per section from ≥ 4 sections spaced 300 μm apart per animal.

Iba-1+ microglial cell bodies containing Hoechst-stained nuclei were counted by two experimenters blinded to genotype and morphine/saline injections. Reliability (Cronbach’s α) of Iba-1+ counts between the two experimenters was assessed for each brain region and the spinal cord ranging between 0.862 and to 0.913 (frontal cortex: *α* = 0.913, hippocampus: *α* = 0.875, striatum: *α* = 0.862, cerebellum: *α* = 0.891, spinal cord: *α* = 0.895). Data presented as the number of Iba-1+ microglia represent the average counts from both experimenters.

Similarly, 3NT+ Iba1+ double-labeled microglia containing Hoechst-stained nuclei were counted by two experimenters blinded to genotype and morphine/saline injections. Reliability (Cronbach’s α) of 3NT+ Iba-1+ microglial counts between the two experimenters was assessed for the striatum (*α* = 0.809) and the spinal cord (*α* = 0.826). The 3-NT+ Iba1+ microglial counts represent the average of both experimenters.

Additionally, Sholl analysis of microglia morphology was performed following previous published studies [92, 93]. Briefly, confocal microscopy was used to collect x 63 z-stack images of the striatum and spinal cord (*n* = 3–4 mice per group/4 sections each) immunodetected for Iba-1 and stained with Hoechst (as outlined above). Maximal intensity projections images were prepared using ZEN 2010 Blue Edition software (Carl Zeiss, Inc., Thornwood, NY) and exported to Fiji build of ImageJ. A total of 4 microglia per animal were individually isolated for analysis per random selection [93]. The soma size was measured in Fiji and the line segment tool was used to draw a line from the center of each soma to the tip of its longest process, providing the maximum branch length (μm). The Sholl analysis plugin software was used to assess additional measures, with the first shell set at 10 μm and subsequent shells set at 5 μm sizes, to determine intersections at each Sholl radius. This provided the critical radius (radius value with the highest number of intersections), the process maximum (the highest number of intersections regardless of radius value), the number of primary processes (intersections at the first Sholl radius), and the process total (total number of intersections).

### Cytokine analysis

Cytokine protein levels were detected in the dorsal striatum and spinal cord by multiplex immunoassay (Mouse Cytokine 23-plex assay kit; #M60009RDPD; Bio-Rad Laboratories, Inc., Hercules, CA, USA) and measured using Bio-Plex 200 plate-reader (Bio-Rad) (Tables 3 and 4). In brief, separate groups of control or Tat transgenic mice (*n* = 5–6 per group) were treated with morphine and/or DOX, samples were dissected, frozen on dry ice, and homogenized in immunoprecipitation (IP) lysis buffer (#87787; Pierce Biotechnology, Rockford, IL, USA) containing an EDTA-free protease/phosphatase inhibitor cocktail (#04693159001; Roche, Mannheim, Germany). Following a bicinchoninic acid assay (#23224; Pierce Biotechnology), protein levels were normalized to 900 μg/mL (striatum) or 500 μg/mL (spinal cord) and run in duplicate on the Bio-Plex assay as per manufacturer’s instructions. Cytokine concentrations were calculated from concurrent standard curves on a five-parameter logistic curve within Bio-Plex Manager 4.0 software.

### Analysis of endocannabinoids and other lipids

Endogenous cannabinoid ligands, including the two main endocannabinoids (eCBs) AEA and 2-AG as well as nine related lipids, including *N*-arachidonoyl glycine (NAGly), peroxisome proliferator activator receptor (PPAR) ligands, such as OEA and PEA, and 2-linoleoyl glycerol (2-LG) were quantified in the dorsal striatum and spinal cord in half of the Tat transgenic mice (*n* = 3–5 per group) after behavioral testing via mass spectrometry (Table 5). Brains were removed immediately following cervical dislocation and decapitation, then the striatum and spinal cord were rapidly dissected, frozen in liquid nitrogen, and stored at − 70 °C until use, as previously described [94]. Samples from the right hemisphere were processed, and substrates were quantified in a manner similar to previous studies [95]. Briefly, frozen striatum and spinal cord samples stored in microfuge tubes were removed from the − 70 °C freezer and immediately placed on dry ice. Each entire sample was then weighed in a TissueLyzer tube with stainless steel ball (QIAGEN, Hilden, Germany), the weight was recorded, and the samples were placed on dry ice until homogenization. Immediately before homogenization, 440 μL of ice-cold methanol (Fisher Scientific, Fair Lawn, NJ), 50 μL of internal standard containing 50 ng/mL 2-AG-d5, 5 ng/mL of AEA-d4, 5 ng/mL of OEA-d4 (Cayman Chemical, Ann Arbor, MI), and 10 μL of 5 mg/mL BHT (Sigma Aldrich, St. Louis, MO) in methanol antioxidant solution was added. Samples were then homogenized at 50 Hz for 2 min (QIAGEN TissueLyzer LT). Immediately after homogenization, samples were placed in a microcentrifuge at 14,000 RPM for 10 min at 4 °C. The supernatant was removed and placed in an MRQ reduced surface activity vial (Microsolv, Leland, NC) and analyzed immediately by liquid chromatography coupled with tandem mass spectrometry [LC/MS/MS; 95]. Immediately after analysis, the remaining supernatant was placed into a − 70 °C freezer and stored until amino acid analysis was performed.

### Amino acid analysis

Various amino acids, including arginine, aspartic acid, glutamic acid, glycine, leucine, phenylalanine, proline, serine, tyrosine, and valine, were quantified from the remaining supernatant of the dorsal striatum and spinal cord after eCB analysis (Table 6). Samples (*n* = 3–5 per group) were processed and substrates quantified as follows; briefly, 10 μL of the striatum or spinal cord homogenate supernatants obtained in the “Analysis of endocannabinoids and other lipids” section were combined with 10 μL of PBS and 10 μL of amino acid internal standard NSK-A (Cambridge Isotope Laboratories, Tewksbury, MA) in 1.5 mL microcentrifuge tubes and placed in a vacuum centrifuge at 55 °C until dry. Samples were then reconstituted with 100 μL of 0.05 N hydrochloric acid and transferred to an MRQ reduced surface activity vial. Amino acids were then analyzed by HILIC-LC/MS/MS as previously described [96] with modifications. Briefly, 1 μL of each sample was injected on an Agilent 2.1 mm × 150 mm, 2.7 μm HILIC-Z column on an Agilent 1260 series high-performance liquid chromatography (HPLC) coupled with an Agilent 6490 triple quadrupole mass spectrometer (Agilent Technologies, Santa Clara, CA). Buffer A consisted of 20 mM ammonium formate in water (pH = 3) and buffer B consisted of 9:1 acetonitrile:water with 20 mM ammonium formate (pH = 3). The HPLC was run with a linear gradient from 0 to 30% buffer A over 10 min at a flow rate of 0.8 mL/min. Mass spectrometric analysis was performed in positive ionization mode. The drying gas was 290 °C at a flow rate of 11 mL/min. The sheath gas was 390 °C at 11 mL/min. The nebulizer pressure was 35 psi. The capillary voltage was 3500 V. Data for amino acids was acquired in dynamic MRM mode using experimentally optimized collision energies obtained by flow injection analysis of authentic standards. Calibration standards for each amino acid were analyzed over a range of concentrations from 0.16 to 100 μM. Calibration curves for each amino acid were constructed using Agilent Masshunter Quantitative Analysis software. Striatum and spinal cord samples were quantitated using the calibration curves to obtain the on-column concentration, followed by multiplication of the results by the appropriate dilution factor to obtain the concentration in pmol/mg of tissue.

### Experimental design

The experimental design is depicted on a timeline in Fig. 1. Tat transgenic mice were exposed to 3 months of DOX treatment to chronically induce Tat expression, which was followed by repeated injections of saline or morphine for 8 days. Body mass was recorded daily. Tat(−) and Tat(+) mice exposed to the escalating morphine doses were imaged on day 8 after morning injections. PET imaging for the control groups was conducted on a different set of Tat(−) and Tat(+) animals that did not receive any injections and were exposed to DOX for 2 weeks. For the behavioral tolerance experiments, antinociceptive tail-flick and hot-plate assays were conducted in the morning of day 9. Brains were harvested immediately after behavioral testing for immunohistochemistry and mass spectrometry analyses. Cytokine analyses were conducted on a separate set of animals.

### Statistical analysis

All data are presented as mean ± the standard error of the mean (SEM). Data sets from body mass, behavioral assays, immunohistochemistry, PET imaging, cytokine assays, and mass spectrometry were analyzed by analysis of variance (ANOVA) with genotype [2 levels: Tat(−) mice, Tat(+) mice] and drug (2 levels: saline, morphine) as between-subjects factors and time or CNS region as a within-subjects factor when appropriate. Violations of compound symmetry in repeated-measures ANOVAs for the within-subjects factors (i.e., comparing time points) were addressed by using the Greenhouse-Geisser degrees (*p*_*GG*_) of freedom correction factor [97]. ANOVAs were followed by Tukey’s post hoc tests when appropriate (SPSS Statistics 25; IBM, Chicago, IL; and/or GraphPad 8.0, San Diego, CA). Thus, differences of α < 0.05 were considered statistically significant. In the tail-flick and hot-plate assays, the dose-response data for %MPE were additionally analyzed for ED_50_ and potency ratios. ED_50_ values were calculated using sigmoidal curvilinear analysis with a variable slope model fixing bottom and top value constraints of 0 and 100, respectively (Prism 8 for Mac OS X; GraphPad Software, La Jolla, CA). The ED_50_ values were considered significantly different if the upper and lower 95% confidence interval (CI) between the dose-response curves did not overlap. Potency ratio values (specifically, ED_50_ shifts) were calculated for parallel curves with fixing bottom and top value constraints of 0 and 100, respectively. A potency-ratio value of greater than 1.0 with a lower 95% CI > 1.0 was considered a significant difference in potency between two dose-response curves (saline versus morphine groups). It should be noted that for the tail-flick assay, values for the short-term morphine-exposed Tat(+) mice did not exceed 50%, thus we are using an estimated ED_50_ value for this group that might not reflect the true ED_50_ value.

## Results

### Body mass and morphine-induced antinociceptive tolerance

**Fig. 2:**
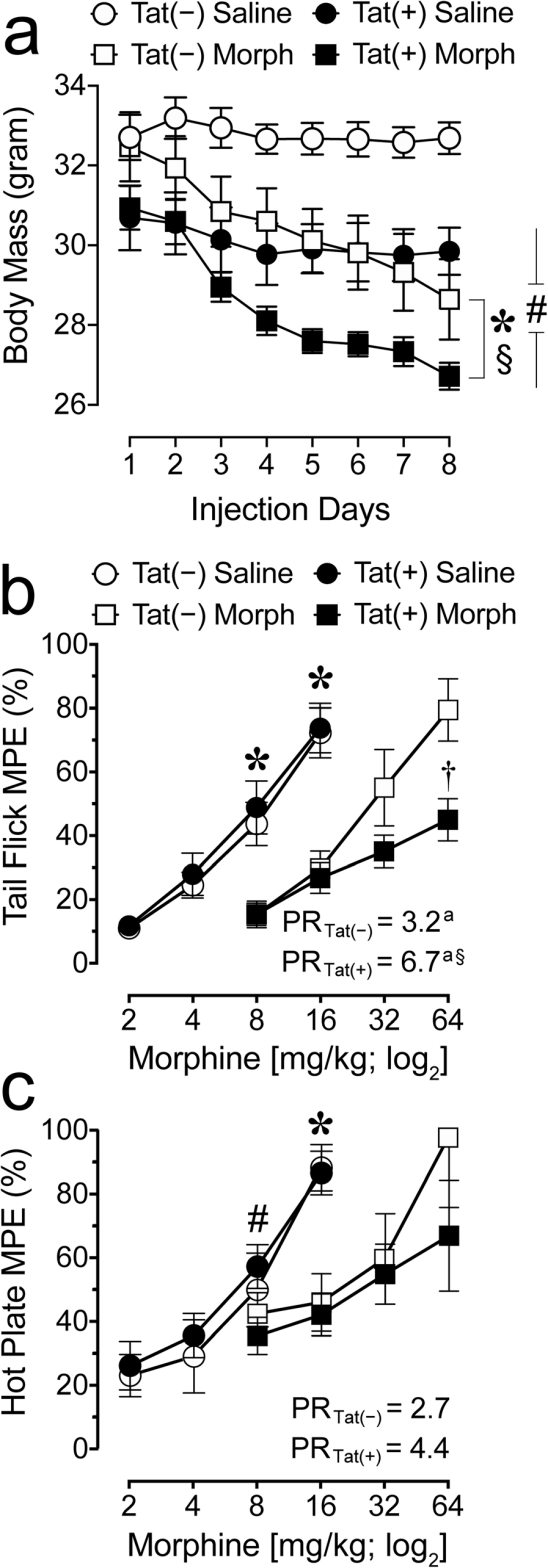
Body mass and behavioral tolerance in Tat transgenic mice were significantly impacted by Tat exposure and morphine treatment. **a** Time course of body mass in Tat(−) and Tat(+) mice exposed to repeated injections of saline or morphine for 8 days. Morphine significantly decreased body mass for Tat(−) and Tat(+) groups overall and across time. Further, Tat expression decreased body mass for Tat(+) mice compared to Tat(−) mice. All data are expressed as mean ± SEM. Statistical significance was assessed by ANOVAs followed by Tukey’s post hoc tests; *p = 0.003 main effect of drug; ^§^*p* < 0.001 time × drug interaction; ^#^*p* < 0.001 main effect of genotype. *Morph* short-term (8-day) morphine injections. *n* = 7–9 mice per group. **b**, **c** Assessment of morphine-induced antinociceptive tolerance in Tat(+) mice compared to Tat(−) mice after morphine or saline exposure for eight days. Each data point represents the mean of the percent maximum possible effect of tail withdrawal (tail flick) or paw withdrawal/lick (hot plate) relative to baseline as a function of acute morphine dose (mg/kg). **b** For the tail-flick assay repeated morphine exposure produced tolerance in Tat(−) and Tat(+) mice compared to their saline-exposed counterparts as indicated by the significant 3.2-fold and 6.7-fold increases in the potency ratios, respectively (^a^*p* < 0.05). Importantly, the 6.7-fold increase in potency for Tat(+) mice was significantly higher than the 3.2-fold increase for Tat(−) mice (based on non-overlapping 95% confidence interval, ^§^*p* < 0.05, see Table 1 for details), indicating increased tolerance for Tat(+) compared to Tat(−) mice when repeatedly exposed to morphine. Note, as the potency ratio of 6.7 is based on an estimated ED_50_ value, caution should be exercised when interpreting the data. Nevertheless, the significant difference between Tat(−) and Tat(+) mice is supported by ANOVA results, specifically in response to an acute morphine injection of 64 mg/kg (^†^*p* = 0.009). **c** For the hot-plate assay morphine-induced tolerance was less robust. ANOVA results indicated significance at 8 mg/kg and/or 16 mg/kg as well as significant differences in ED_50_ values for Tat(+) mice exposed to saline versus morphine (based on non-overlapping 95% confidence interval, see Table 1 for details). However, the 2.7- and 4.4-fold increase in potency ratio for morphine exposure in Tat(−) and Tat(+) mice, respectively, was not significant. All data are expressed as mean ± SEM. Statistical significance was assessed by ANOVA followed by Tukey’s post hoc test; *p < 0.05 vs. 8 or 16 mg/kg acute morphine administration in the corresponding Tat mouse group exposed to repeated morphine; for Tat(+) mice: ^#^*p* < 0.05 vs. Tat(+) mice exposed to repeated morphine. “PR” = potency ratio, as determined by the ED_50_ shift between the saline and morphine-exposed groups (see text for details), ^a^*p* < 0.05; ^§^*p* < 0.05 vs. 3.2-fold PR for Tat(−) mice. *Morph* short-term (8-day) morphine injections, *MPE* maximun possible effect, *PR* potency ratio. Tail flick, *n* = 7–9 mice per group; hot plate, *n* = 3–4 mice per group

Body mass was recorded daily throughout the 8-day repeated injection regimen (Fig. 2a). A three-way mixed ANOVA with time as a within-subjects factor (using Greenhouse-Geisser degrees (*p*_*GG*_) of freedom as a correction factor [97]) and genotype and drug as between-subjects factors was conducted. A significant main effect of time [*F*(7, 196) = 65.6, *p*_*GG*_ < 0.001] and time × drug interaction [*F*(7, 196) = 34.0, *p*_*GG*_ < 0.001] were noted, with a significant reduction in body mass over injection days for morphine-treated groups [Tat(−) mice, *F*(7, 42) = 35.0, *p*_*GG*_ < 0.001; Tat(+) mice, *F*(7, 56) = 48.1, *p*_*GG*_ < 0.001], but not saline-exposed Tat(−) and Tat(+) groups. This morphine-induced decline in body mass was confirmed statistically by an overall main effect of drug [*F*(1, 28) = 10.8, *p* = 0.003]. Further, a significant main effect of genotype was noted [*F*(1, 28) = 15.8, *p* < 0.001], with Tat(+) mice demonstrating lower body mass compared to Tat(−) mice independent of drug exposure.

The antinociceptive effects of morphine were determined using the tail-flick and hot-plate assays (Fig. 2b, c). All groups, except the Tat(−) group in the hot-plate test, demonstrated tolerance following the 8-day repeated escalating morphine dosing regimen compared to saline-treated mice in both assays (Table 1). The repeated morphine dosing regimen led to rightward shifts in the acute morphine dose-response curves in Tat(−) and Tat(+) mice compared to saline exposure, as indicated by significant differences in the ED_50_ values (based on non-overlapping 95% CI) with potency ratio values varying between 3.2- and 6.7-fold (Table 1). Note that potency ratios for the hot-plate test did not reveal significance in Tat(−) and Tat(+) mice, potentially due to the small sample size that increased the likelihood of type II error. Additional ANOVA results further support the notion of induced tolerance by morphine with significant decreased %MPE in the tail-flick assay for repeated morphine-exposed mice compared to mice receiving saline after acute cumulative morphine injections of 8 mg/kg [Tat(−), *F*(1, 13) = 11.6, *p* = 0.005, Tat(+), *F*(1, 15) = 14.9, *p* = 0.002] and 16 mg/kg [Tat(−), *F*(1, 13) = 18.7, *p* = 0.001, Tat(+), *F*(1, 15) = 27.9, *p* < 0.001; Fig. 2b]. Similar effects were noted for the hot-plate assay, with ANOVA results demonstrating significant decreased %MPE values in repeated morphine-exposed mice after the 8 mg/kg acute cumulative morphine dose injection compared to mice receiving saline [Tat(+), *F*(1, 6) = 5.8, *p* = 0.052] and 16 mg/kg [Tat(−), *F*(1, 5) = 11.9, *p* = 0.018, Tat(+), *F*(1, 6) = 22.1, *p* = 0.003; Fig. 2c]. Importantly, an increased tolerance is noted by Tat induction in the tail-flick assay with the estimated ED_50_ value for morphine-treated Tat(+) mice significantly differing from the ED_50_ value for the morphine-exposed Tat(−) mice (based on non-overlapping 95% CI, see Table 1). Further, when exposed to repeated escalating morphine, Tat(+) mice displayed decreased tail-flick %MPE (44.9 ± 6.7) compared to Tat(−) mice (79.5 ± 9.7) in response to an acute injection of 64 mg/kg morphine (tail flick, *F*(1, 14) = 9.2, ^†^*p* = 0.009, Fig. 2b), supporting the notion that Tat increased the tolerance to repeated morphine exposure. No other significant effects were noted. Together, these results support previous tail flick data showing that Tat expression increases morphine tolerance for the tail-flick task [98] with less robust changes in the supraspinal-related hot-plate assay.

**Table 1:** Morphine-induced antinociceptive tolerance (%MPE) in the tail-flick (mean ± SEM, *n* = 7–9 per group) and hot-plate (mean ± SEM, *n* = 3–4 per group) assays

Assay	Mouse	Repeated saline ED_50_ (95% CI)	Repeated morphine ED_50_ (95% CI)	Sig.	Potency ratio (95% CI)
Tail flick	Tat(−)	8.6 (7.0–10.5)	26.6 (20.3–34.8)	*	3.2 (2.3–4.4)^a^
	Tat(+)	7.8 (6.2–9.8)	91.3 (60.7–137.4)^1^	*	6.7 (4.6–10.8)^a^^§^
Hot plate	Tat(−)	6.1 (4.3–8.7)	14.5 (8.1–26.1)	n.s.	2.7 (0.5–8.7)
	Tat(+)	5.4 (4.2–7.0)	23.0 (11.4–46.3)	*	4.4 (0.7–28.9)

ED50 values (mg/kg) and potency ratio values are derived from acute cumulative dose-response curves obtained for repeated short-term saline- and morphine-exposed mice.; *n*.*s*. not significant; *p < 0.05 saline vs. corresponding morphine group; ^a^*p* < 0.05; ^§^Indicates significance from Tat(−) in the tail-flick assay based on non-overlapping 95% CI. Note, as the potency ratio of 6.7 is based on an estimated ED_50_ value caution should be exercised when interpreting the data. *CI* confidence interval

^1^Estimated ED_50_

### PET [^18^F]-PBR111 neuroinflammation ligand

**Fig. 3:**
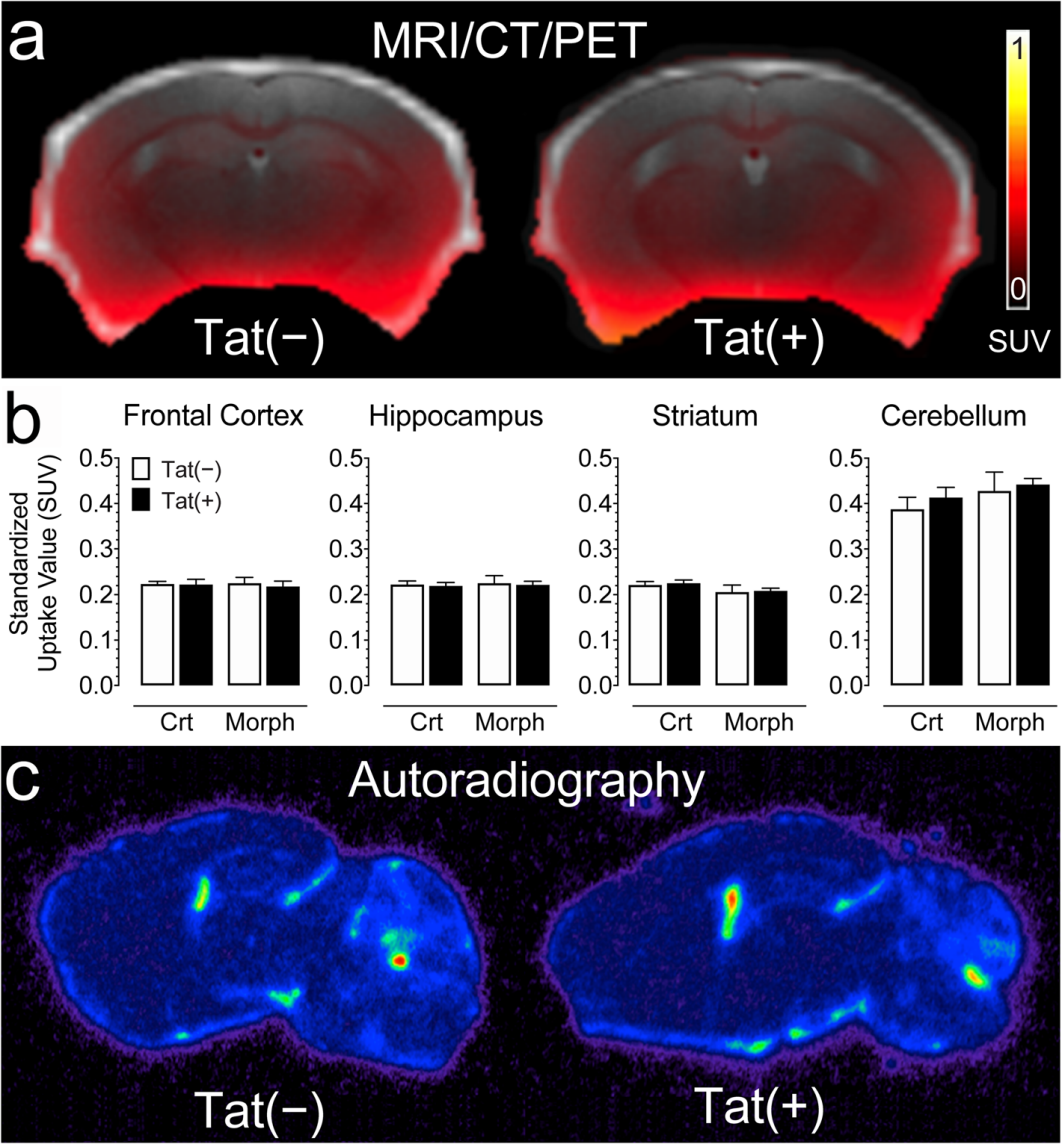
No significant changes were noted for neuroinflammation assessed by in vivo PET imaging using the [^18^F]-PRB111 probe in Tat transgenic mice in the presence or absence of morphine treatment. **a** A representative overlay image of a Tat(−) and Tat(+) mice show MR (gray), CT (white), and PET (SUV scale, yellow-red) imaging in the coronal plane. **b** PET imaging of Tat transgenic mice demonstrates the successful uptake of the [^18^F]-PBR111 radiotracer in different brain regions. No significant effect was noted for drug and/or genotype. **c** A representative brain image of a Tat(−) and Tat(+) mouse shows similar distribution of [^18^F]-PBR111 binding via post-mortem autoradiography in both groups in the parasagittal plane approximately 2 mm from the midline. All data are expressed as mean ± SEM. Statistical significance was assessed by ANOVA followed by Tukey’s post hoc test. *SUV* standardized uptake value, *Crt* no injections, *Morph* short-term (8-day) morphine injections. *n* = 5–7 mice per group

Non-invasive PET imaging using the [^18^F]-PRB111 probe was conducted to monitor active TSPO inflammatory processes in the brains of Tat transgenic mice in vivo (*n* = 5–7 per group; Fig. 3). Uptake of the [^18^F]-PBR111 radiotracer was measured in different brain regions, including the frontal cortex, hippocampus, striatum, and cerebellum for the following groups: control Tat(−) mice and Tat(+) mice, and Tat(−) and Tat(+) mice exposed to morphine (Fig. 3b). A three-way mixed ANOVA with brain region as a within-subjects factor and genotype and drug as between-subjects factors was conducted. Brain region significantly affected [^18^F]-PBR111 uptake [*F*(3, 60) = 256.4, *p*_*GG*_ < 0.001] with higher levels noted in the cerebellum compared to any other brain region (*p*’s < 0.001). No other effect was significant, indicating that genotype and drug did not affect brain inflammation assessed by the [^18^F]-PRB111 imaging probe, which was confirmed by post-mortem autoradiography (Fig. 3c).

TPSO is widely expressed by tissues outside the CNS, including muscle and connective tissue [99, 100] creating artifactually high [^18^F]-PRB111 signals in the ventral forebrain, cerebellum, and spinal cord. The high signal intensity breaching into the ventral forebrain was mainly due to the signals outside the brain (Fig. 3a), while in the cerebellum artificially high signals were caused by the extensive infolding of meningeal tissue, e.g., the tentorium cerebelli. The spinal cord was not examined (i) because of the strong partial-volume effects resulting from artifactually increased [^18^F]-PRB111 signals from surrounding muscle compared to the small volume of spinal cord and (ii) because of the limited field of view of the scanner (47 mm in transaxial length), which is unable to image the brain and spinal cord concurrently. Accordingly, TSPO was not quantified in some ventral forebrain regions, the cerebellum, or spinal cord. Overall, no effects were noted for exposure to Tat and/or morphine on neuroinflammation assessed by PET imaging of [^18^F]-PBR111 uptake.

### Immunohistochemistry

#### Quantification of number of Iba-1+ microglia in 5 CNS regions

**Fig. 4:**
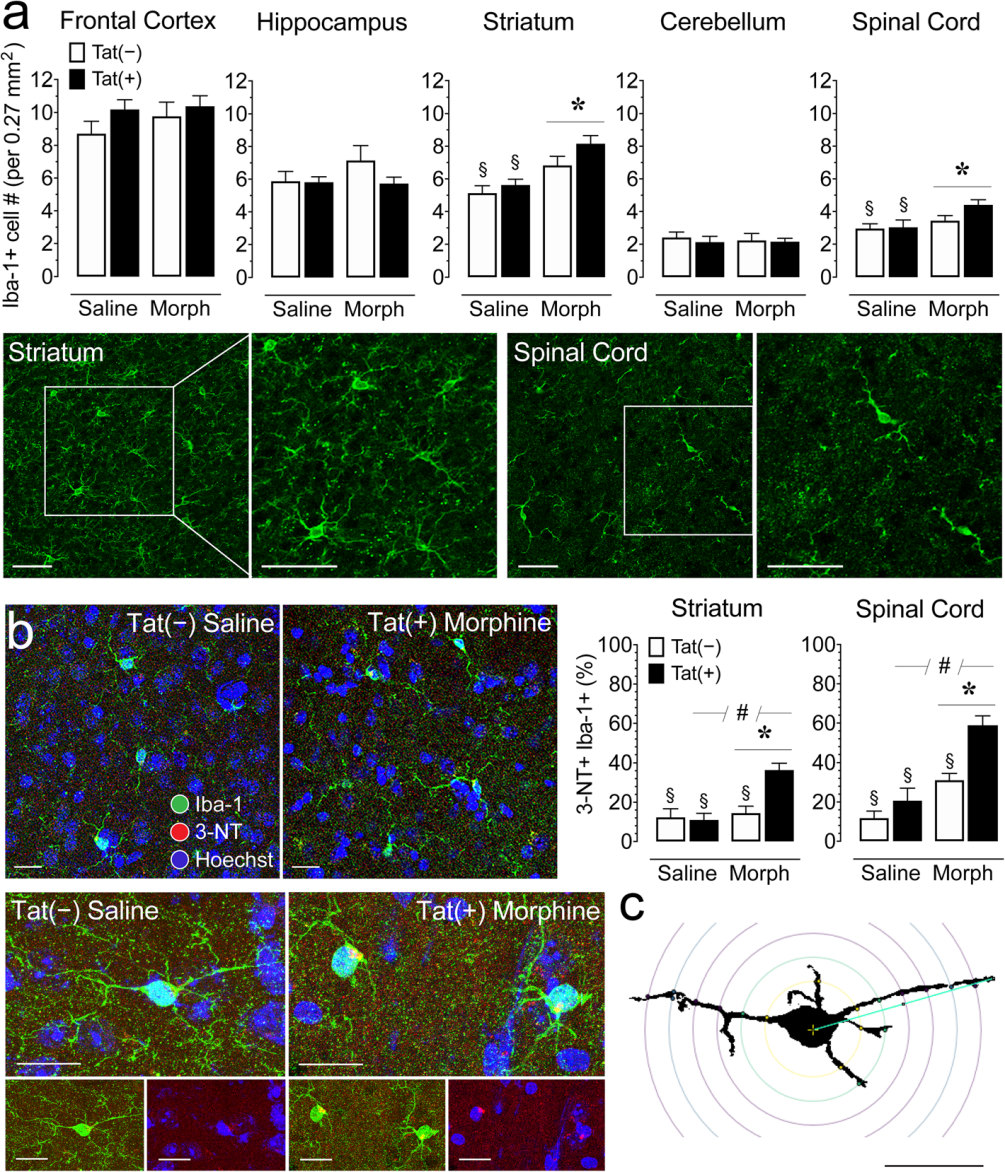
Morphine treatment increased the number of microglia (Iba-1+) and morphine and Tat exposure increased activated microglia (3-NT+ Iba-1+) in the striatum and spinal cord of Tat transgenic mice. **a** Quantification of Iba-1+ cell counts in 5 different CNS regions, including the frontal cortex, hippocampus, striatum, cerebellum, and spinal cord were examined for Iba-1+ cells. Morphine upregulated Iba-1+ cell counts in the striatum and spinal cord, specifically for the morphine-treated, Tat(+) mice. Tat expression showed no significant effect. CNS sections from the striatum and spinal cord stained for Iba-1+ cells (green) taken at × 20 with a higher magnification images taken at × 63. Scale bars = 50 μm. **b** Immunofluorescent images were taken in the spinal cord at × 63 and show double-labeling for the microglial marker Iba-1 (green) and 3-nitrotyrosine (3-NT; red), a reactive nitrosyl product detected in activated microglia, with containing Hoechst-stained nuclei. The 3-NT+ Iba1+ double-labeled microglia from a saline-treated Tat(−) mouse is ramified and shows relative low level of 3-NT expression. In contrast the 3-NT+ Iba1+ double-labeled microglia from the morphine-treated Tat(+) mouse shows decreased processes and the level of 3-NT is noticeably higher (see Table 2 for quantification via Sholl analysis). Scale bars = 20 μm. Percent 3-NT+ Iba-1+ cell counts based on total number of Iba-1 cells was quantified in the striatum and spinal cord. Morphine and Tat expression increased 3-NT+ Iba-1+ cell counts (%) in the striatum and spinal cord, specifically for morphine-treated Tat(+) mice. **c** Example of a single microglial cell with concentric Sholl radii (purple circles) superimposed on the image. Scale bar = 20 μm. All data are expressed as mean ± SEM. Statistical significance was assessed by ANOVA followed by Tukey’s post hoc test; *p < 0.05 main effect of drug; ^#^*p* < 0.05 main effect of genotype; ^§^*p* < 0.05 vs. morphine-exposed Tat(+) mice. *Morph* short-term (8-day) morphine injections. *n* = 3–4 mice per group/4 sections each

Immunohistochemistry experiments were conducted to assess microglia by quantifying the number of Iba-1+ immunoreactive microglia in various brain regions and the spinal cord (*n* = 3–4 per group/4 sections each; Fig. 4a). A three-way mixed ANOVA with CNS region as a within-subjects factor and genotype and drug as between-subjects factors was conducted and revealed significant effects. Number of microglia differed based on CNS region [*F*(4, 196) = 129.3, *p* < 0.001] with all CNS regions differing from each other (*p*’s < 0.001), except for the hippocampus compared to the striatum. Additionally, morphine increased the number of microglia [*F*(1, 49) = 10.0, *p* = 0.003] and significantly interacted across CNS regions [*F*(4, 196) = 2.6, *p* = 0.040]. To assess this interaction further, separate two-way ANOVAs for the four brain regions and spinal cord were conducted and revealed significant effects only for the striatum and spinal cord. Morphine significantly increased the number of Iba-1+ cells in the striatum [*F*(1, 49) = 18.9, *p* < 0.001] and spinal cord [*F*(1, 49) = 4.7, *p* = 0.036]. Morphine-treated Tat(+) mice showed increased Iba1+ microglial accumulation compared to saline-treated, Tat(+) mice (striatum, *p* = 0.002; spinal cord, *p* = 0.043), and Tat(−) mice (striatum, *p* < 0.001; spinal cord, *p* = 0.049; Fig. 4a).

#### Quantification of activated 3-NT+ Iba-1+ microglia in the striatum and spinal cord

Immunohistochemistry experiments were conducted to assess activated microglia by quantifying the number of 3-NT+ Iba-1+ double-labeled cells in the striatum and spinal cord (*n* = 3–4 per group/4 sections each; Fig. 4b). Separate two-way ANOVAs for the striatum and spinal cord with genotype and drug as between-subjects factors were conducted and revealed significant effects for both regions. Morphine treatment significantly increased the percentage of 3-NT+ Iba-1+ microglia in the striatum [*F*(1, 55) = 13.9, *p* < 0.001] and spinal cord [*F*(1, 56) = 32.5, *p* < 0.001], which was also noted for Tat expression [striatum, *F*(1, 55) = 7.7, *p* = 0.008; spinal cord, *F*(1, 56) = 13.1, *p* = 0.001]. Morphine and Tat treatments significantly interacted to increase the percentage of 3-NT+ Iba-1+ co-localized microglia within the striatum [*F*(1, 55) = 9.9, *p* = 0.003] and a similar trend was noted for the spinal cord [*F*(1, 56) = 3.4, *p* = 0.070]. Specifically, morphine-treated, sections from Tat(+) mice striatal, and spinal cord showed increased 3-NT+ Iba-1+ microglia compared to sections from Tat(−) mice (striatum, *p* < 0.001; spinal cord, *p* = 0.001), as well as compared to sections from saline-treated, Tat(−) mice striatum (*p* < 0.001), and spinal cord (*p* < 0.001), and Tat(+) mice striatum (*p* < 0.001) and spinal cord (*p* < 0.001). Together, these data indicate that increased drug tolerance was accompanied by morphine-dependent increases in 3-NT+ Iba-1+ immunoreactive microglia.

#### Sholl analysis of microglia morphology in the striatum and spinal cord

Sholl analyses were conducted to assess microglia morphology in the striatum and spinal cord (*n* = 3–4 per group/4 sections each; Table 2). Separate two-way ANOVAs for the striatum and spinal cord with genotype and drug as between-subjects factors were conducted and revealed significant effects for only the spinal cord. Tat treatment significantly enlarged the soma area (Table 2) which is associated with amoeboid morphology [92]. Additionally, morphine significantly interacted with Tat in the spinal cord to decrease number of primary processes (intersections at the first Sholl radius), process maximum (the highest number of intersections regardless of radius value), and process total (total number of intersections) but Tukey’s post hoc test revealed no significant differences between groups for any of the three measures. Nevertheless, overall morphine exposure in the presence of Tat expression appears to induce abnormal morphology specifically in the spinal cord.

**Table 2:** Effects of Tat and morphine on microglial morphology in the striatum and spinal cord of Tat transgenic mice

CNS region	Measure	Genotype	Repeated saline	Repeated morphine	Genotype effect		Drug effect		Genotype x drug	
			mean ± SEM	mean ± SEM	*F*_1, 56_	*p*	*F*_1, 56_	*p*	*F*_1, 56_	*p*
Striatum	Soma area (μm^2^)	Tat(−)	51.1 ± 2.16	46.7 ± 2.98	< 1.0	0.49	2.9	0.09	< 1.0	0.96
		Tat(+)	49.2 ± 2.41	45.1 ± 2.00						
	Maximum branch length (μm)	Tat(−)	35.3 ± 3.32	32.5 ± 2.59	< 1.0	0.59	< 1.0	0.84	1.7	0.20
		Tat(+)	30.7 ± 1.90	34.4 ± 2.19						
	Critical radius (μm)	Tat(−)	14.6 ± 1.56	13.4 ± 1.35	< 1.0	0.51	< 1.0	0.97	< 1.0	0.63
		Tat(+)	14.4 ± 1.76	15.6 ± 1.20						
	Number of primary process	Tat(−)	4.8 ± 0.56	4.1 ± 0.30	2.8	0.10	< 1.0	0.92	2.8	0.10
		Tat(+)	3.5 ± 0.29	4.1 ± 0.45						
	Process maximum	Tat(−)	6.8 ± 0.83	5.3 ± 0.60	< 1.0	0.93	< 1.0	0.70	2.3	0.13
		Tat(+)	5.5 ± 1.00	6.4 ± 0.77						
	Process total	Tat(−)	21.5 ± 3.02	16.1 ± 2.20	< 1.0	0.89	< 1.0	0.70	2.1	0.16
		Tat(+)	16.9 ± 3.49	19.9 ± 2.77						
Spinal cord	Soma area (μm^2^)	Tat(−)	48.5 ± 3.42	50.9 ± 3.79	**4.2**	**0.04**	3.8	0.06	1.5	0.23
		Tat(+)	51.4 ± 2.45	62.2 ± 3.73						
	Maximum branch length (μm)	Tat(−)	35.1 ± 2.59	36.2 ± 2.71	< 1.0	0.51	< 1.0	0.66	< 1.0	0.98
		Tat(+)	33.4 ± 2.32	34.6 ± 2.19						
	Critical radius (μm)	Tat(−)	16.7 ± 1.98	15.6 ± 1.20	< 1.0	0.44	< 1.0	0.98	< 1.0	0.61
		Tat(+)	17.2 ± 2.28	18.1 ± 2.04						
	Number of primary process	Tat(−)	2.3 ± 0.28	2.7 ± 0.33	< 1.0	0.81	1.5	0.23	**5.0**	**0.03**
		Tat(+)	3.2 ± 0.47	2.0 ± 0.20						
	Process maximum	Tat(−)	3.6 ± 0.31	4.6 ± 0.57	< 1.0	0.81	< 1.0	0.78	**5.4**	**0.02**
		Tat(+)	4.8 ± 0.58	3.6 ± 0.27						
	Process total	Tat(−)	11.5 ± 1.56	15.7 ± 2.30	< 1.0	0.58	< 1.0	0.95	**5.1**	**0.03**
		Tat(+)	14.6 ± 1.75	10.6 ± 1.22						

Sholl analysis of microglial morphology in the striatum and spinal cord of repeated (8-day) saline- or morphine-treated Tat(−) and Tat(+) mice. Data are expressed as the mean ± SEM. The parameters measured by Sholl analysis are indicated in parentheses in the second column. One-way ANOVAs for each CNS region were conducted with genotype and drug as between-subjects factors. *F* values and *p* values are presented from ANOVA results. Bolded values denote significant differences at *α* = 0.05; mean ± SEM, *n* = 3–4 mice per group/4 sections each

### Cytokine production in the striatum and spinal cord

**Fig. 5:**
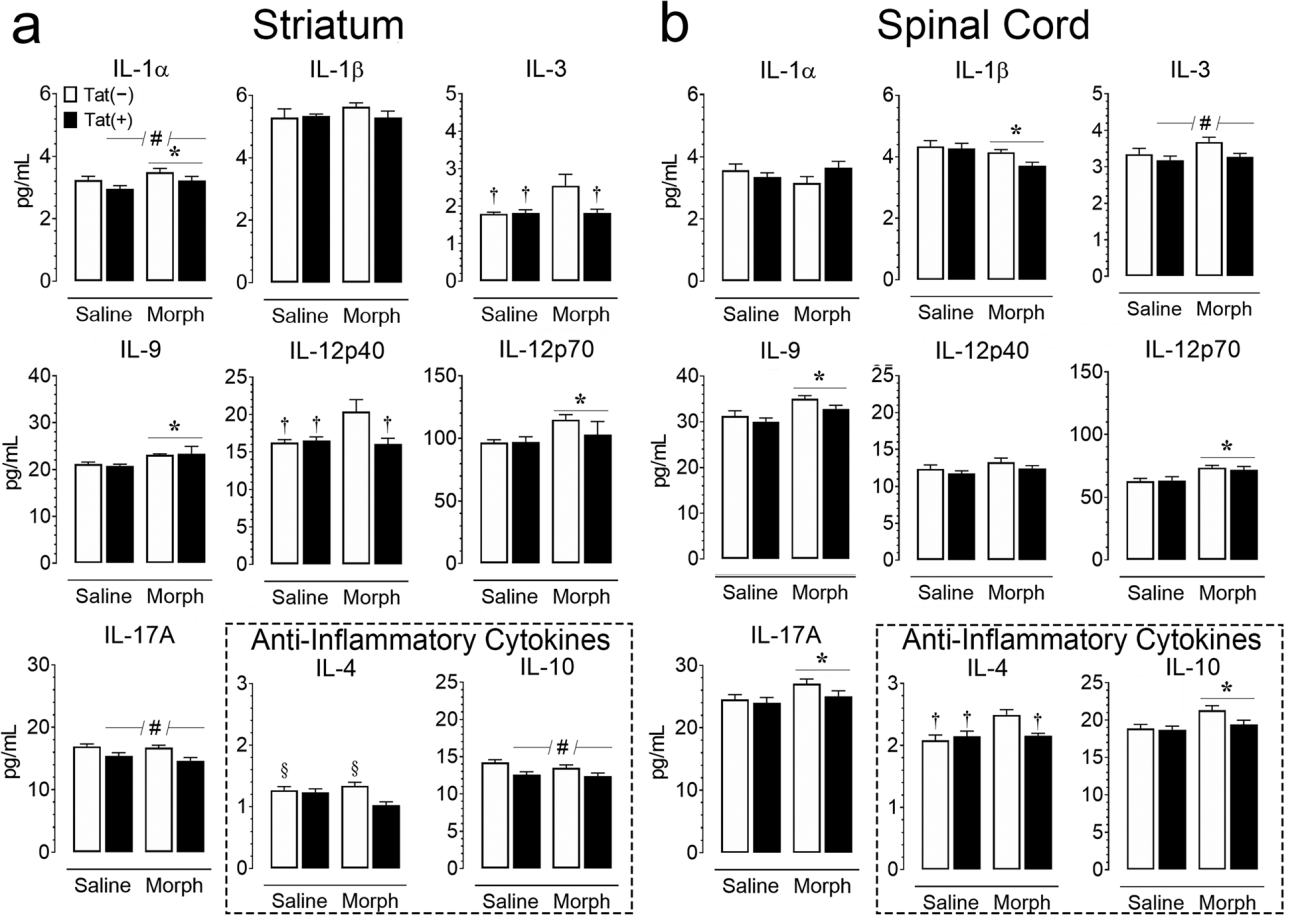
Cytokine concentrations (pg/mL) are impacted by Tat exposure and morphine in the striatum (**a**) and spinal cord (**b**) of Tat transgenic mice. Proinflammatory cytokines showed morphine- and Tat-specific effects in the striatum and spinal cord. Morphine treatment led to a significant increase in multiple proinflammatory cytokines in the striatum (IL-1α, IL-9, IL-12p70) and spinal cord (IL-9, IL-12p70, IL-17A), while reducing IL-1β in the spinal cord. Tat exposure reduced several cytokines in the striatum (IL-1α, IL-17A) and spinal cord (IL-3). Morphine and Tat interactions were noted for IL-3 and IL-12p40 in the striatum. Anti-inflammatory cytokine IL-10 (highlighted, bottom right) was elevated by morphine in the spinal cord and reduced by Tat expression in the striatum, whereas IL-4 indicated a significant morphine by Tat interaction for both CNS regions. All data are expressed as mean pg/mL ± SEM. Samples were normalized to 900 μg/mL (striatum) or 500 μg/mL (spinal cord) via BCA. Statistical significance was assessed by ANOVA followed by Tukey’s post hoc test; *p < 0.05 main effect of drug; ^#^*p* < 0.05 main effect of genotype; ^†^*p* < 0.05 vs. morphine-exposed Tat(−) mice; ^§^*p* < 0.05 vs. morphine-exposed Tat(+) mice. *Morph* short-term (8-day) morphine injections. *n* = 5–6 mice per group

**Fig. 6:**
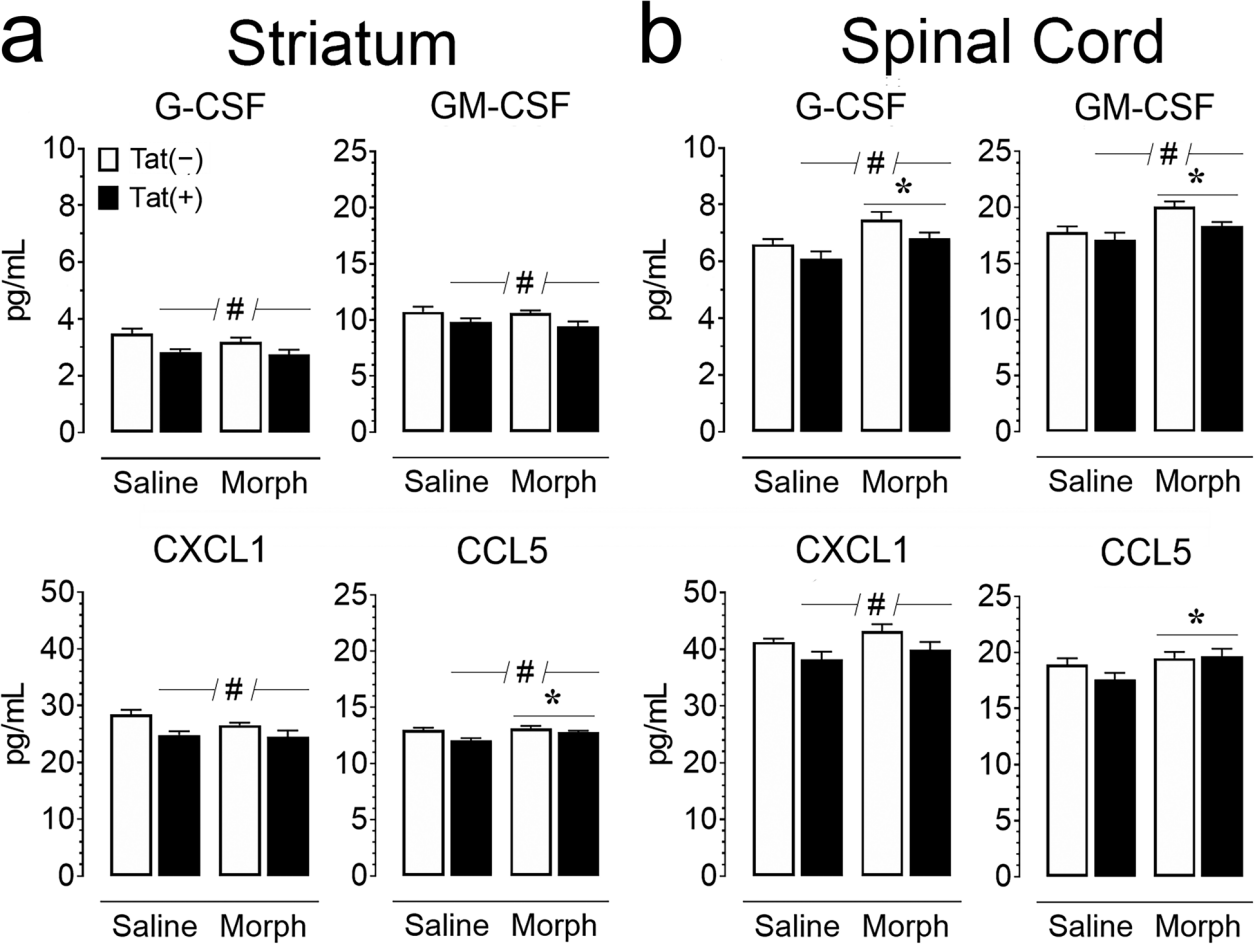
Chemokine levels (pg/mL) are altered by Tat exposure or morphine in the striatum (**a**) and spinal cord (**b**) of Tat transgenic mice. Concentrations of chemokines were assessed using a Bio-Plex cytokine array. G-CSF, GM-CSF, and CXCL1 showed significant Tat-induced reductions in both regions, while CCL5 was only reduced in striatum (^#^*p* < 0.05). Morphine treatment increased G-CSF and GM-CSF concentrations within the spinal cord, and CCL5 concentrations in both regions. All data are expressed as mean pg/mL ± SEM. Samples were normalized to 900 μg/mL (striatum) or 500 μg/mL (spinal cord) via BCA. Statistical significance was assessed by ANOVA followed by Tukey’s post hoc test; *p < 0.05 main effect of drug; ^#^*p* < 0.05 main effect of genotype. *Morph* short-term (8-day) morphine injections. *n* = 5–6 mice per group

Protein levels of proinflammatory, anti-inflammatory, and chemotactic cytokines were measured using a Bio-Plex 23-plex multiplexed immunoassay within the striatum and spinal cord tissue lysate (*n* = 5–6 per group; Tables 3 and 4; Figs. 5 and 6). Cytokine levels for the striatum and spinal cord were mixed but indicated mostly higher levels in the spinal cord compared to the striatum. A three-way mixed ANOVA with CNS region as a within-subjects factor and genotype and drug as between-subjects factors indicated a significant main effect of CNS region (*p* < 0.05) for all measured cytokines except IL-13, with the spinal cord generally exhibiting an increase in proinflammatory, anti-inflammatory, and chemotactic cytokines. To assess Tat and morphine’s effect for each CNS region, separate two-way ANOVAs for the striatum and spinal cord with genotype and drug as between-subjects factors were conducted for each cytokine, and are summarized in Figs. 5 and 6, with additional cytokine measures being summarized in Tables 3 and 4.

**Table 3:** Effects of Tat and morphine on cytokine concentrations in the striatum of Tat transgenic mice that are not presented in Figs. 5 and 6

Striatal cytokines	Genotype	Repeated saline	Repeated morphine	Genotype effect		drug effect		Genotype x drug	
pg/mL		mean ± SEM	mean ± SEM	*F*_1, 19_	*p*	*F*_1, 19_	*p*	*F*_1, 19_	*p*
*Proinflammatory Cytokines*									
IL-2	Tat(−)	10.7 ± 0.61	10.7 ± 0.18	< 1.0	0.59	< 1.0	0.48	< 1.0	0.50
	Tat(+)	10.6 ± 0.33	11.2 ± 0.12						
IL-6	Tat(−)	2.5 ± 0.08	3.2 ± 0.13	1.6	0.22	**33.0**	< **0.01**	1.7	0.20
	Tat(+)	2.5 ± 0.08	2.9 ± 0.13						
IFN-γ	Tat(−)	24.3 ± 0.63	24.9 ± 0.26	3.3	0.08	1.9	0.18	< 1.0	0.60
	Tat(+)	22.6 ± 0.46	24.0 ± 1.40						
TNF-α	Tat(−)	45.1 ± 1.33	48.3 ± 1.58	< 1.0	0.87	2.9	0.11	< 1.0	0.68
	Tat(+)	44.4 ± 1.47	49.9 ± 5.14						
*Anti-Inflammatory Cytokines*									
IL-5	Tat(−)	2.2 ± 0.15	2.8 ± 0.09	1.7	0.20	**26.0**	< **0.01**	< 1.0	0.99
	Tat(+)	2.0 ± 0.08	2.7 ± 0.20						
IL-13	Tat(−)	36.9 ± 1.79	48.1 ± 0.99	3.4	0.08	**39.6**	< **0.01**	< 1.0	0.54
	Tat(+)	31.9 ± 0.93	45.7 ± 3.73						
*Chemokines*									
CCL2	Tat(−)	42.2 ± 1.36	42.3 ± 1.08	**13.6**	< **0.01**	< 1.0	0.60	< 1.0	0.60
	Tat(+)	38.4 ± 1.01	37.1 ± 1.38						
CCL3	Tat(−)	8.3 ± 0.82	10.5 ± 1.08	< 1.0	0.66	3.2	0.09	< 1.0	0.45
	Tat(+)	8.6 ± 0.63	9.5 ± 0.73						
CCL4	Tat(−)	48.8 ± 0.42	47.8 ± 0.84	**18.4**	< **0.01**	3.4	0.08	< 1.0	0.61
	Tat(+)	46.0 ± 0.93	44.2 ± 0.70						
CCL11	Tat(−)	99.9 ± 1.77	96.6 ± 1.24	2.2	0.15	1.3	0.27	< 1.0	0.85
	Tat(+)	95.5 ± 3.53	91.0 ± 6.01						

Cytokine concentrations in the striatum of repeated (8-day) saline- or morphine-treated Tat(−) and Tat(+) mice. Data are expressed as mean ± SEM in pg/mL. Samples were loaded at 900 μg/mL total protein. Two-way ANOVAs for each CNS region were conducted with genotype and drug as between-subjects factors. *F* values and *p* values are presented from ANOVA results. Bolded values denote significant differences at *α* = 0.05; *n* = 5–6 mice per group. Additional cytokines and chemokines are represented in Figs. 5 and 6

**Table 4:** Effects of Tat and morphine on cytokine concentrations in the spinal cord of Tat transgenic mice that are not presented in Figs. 5 and 6

Spinal cord cytokines	Genotype	Repeated saline	Repeated morphine	Genotype effect		Drug effect		Genotype x drug	
pg/mL		mean ± SEM	mean ± SEM	*F*_1, 19_	*p*	*F*_1, 19_	*p*	*F*_1, 19_	*p*
Proinflammatory cytokines									
IL-2	Tat(−)	12.5 ± 0.36	14.3 ± 0.47	2.3	0.15	**16.9**	< **0.01**	< 1.0	0.98
	Tat(+)	11.8 ± 0.44	13.6 ± 0.46						
IL-6	Tat(−)	3.4 ± 0.17	3.6 ± 0.19	1.5	0.24	1.5	0.23	< 1.0	0.79
	Tat(+)	3.2 ± 0.13	3.4 ± 0.19						
IFN-γ	Tat(−)	29.1 ± 0.76	30.3 ± 0.71	< 1.0	0.38	**6.2**	**0.02**	< 1.0	0.37
	Tat(+)	27.8 ± 0.74	30.3 ± 0.76						
TNF-α	Tat(−)	52.4 ± 1.70	51.8 ± 1.67	1.6	0.22	1.6	0.22	2.8	0.11
	Tat(+)	47.8 ± 1.73	52.4 ± 0.48						
Anti-inflammatory cytokines									
IL-5	Tat(−)	3.4 ± 0.22	3.4 ± 0.12	**4.3**	**0.05**	< 1.0	0.44	< 1.0	0.36
	Tat(+)	3.2 ± 0.13	2.9 ± 0.11						
IL-13	Tat(−)	40.8 ± 3.86	41.6 ± 3.27	1.6	0.21	1.7	0.20	2.4	0.14
	Tat(+)	41.7 ± 2.73	32.4 ± 2.63						
Chemokines									
CCL2	Tat(−)	50.8 ± 1.13	57.7 ± 1.66	2.4	0.14	**12.7**	< **0.01**	< 1.0	0.64
	Tat(+)	48.9 ± 1.83	54.2 ± 2.24						
CCL3	Tat(−)	5.1 ± 0.14	5.3 ± 0.19	3.5	0.08	< 1.0	0.71	< 1.0	0.53
	Tat(+)	4.8 ± 0.21	4.8 ± 0.21						
CCL4	Tat(−)	67.4 ± 1.27	74.5 ± 0.78	1.4	0.25	**20.3**	< **0.01**	< 1.0	0.44
	Tat(+)	66.9 ± 1.85	71.9 ± 1.18						
CCL11	Tat(−)	108.4 ± 5.37	100.4 ± 4.32	< 1.0	0.57	< 1.0	0.72	1.7	0.20
	Tat(+)	99.3 ± 4.37	103.9 ± 5.01						

Cytokine concentrations in the spinal cord of repeated (8-day) saline- or morphine-treated Tat(−) and Tat(+) mice. Data are expressed as mean ± SEM in pg/mL. Samples were loaded at 500 μg/mL total protein. Two-way ANOVAs for each CNS region were conducted with genotype and drug as between-subjects factors. *F* values and *p* values are presented from ANOVA results. Bolded values denote significant differences at *α* = 0.05; *n* = 5–6 mice per group. Additional cytokines and chemokines are represented in Figs. 5 and 6

Within proinflammatory cytokines (Tables 3 & 4, Fig. 5), Tat expression reduced striatal concentrations of IL-1α [*F*(1, 19) = 5.3, *p* = 0.033] and IL-17A [*F*(1, 19) = 15.6, *p* = 0.001]. IL-3 protein levels were reduced in the spinal cord of Tat (+) mice [*F*(1, 19) = 5.0, *p* = 0.038]. Morphine and Tat treatments significantly interacted to reduce concentrations of inflammatory mediators IL-3 [*F*(1, 19) = 4.5, *p* = 0.047] and IL-12p40 [*F*(1, 19) = 5.889, *p* = 0.025] within the striatum. Morphine-treated, Tat(−) striatal samples showed elevated IL-3 and IL-12p40 compared to saline-treated, Tat(−) (IL-3, *p* = 0.028; IL-12p40, *p* = 0.025) and Tat(+) (IL-3, *p* = 0.033; IL-12p40, *p* = 0.038), as well as morphine-treated, Tat(+) mice (IL-3, *p* = 0.046; IL-12p40, *p* = 0.024). In contrast, morphine exposure correlated with increased protein concentrations of IL-1α [*F*(1, 19) = 4.677, *p* = 0.044], IL-6 [*F*(1, 19) = 33.0, *p* < 0.001], IL-9 [*F*(1, 19) = 8.7, *p* = 0.008] and IL-12p70 [*F*(1, 19) = 4.7, *p* = 0.043] within the striatum. Within the spinal cord, morphine significantly reduced IL-1β [*F*(1, 19) = 5.9, *p* = 0.025], but increased concentrations of IL-2 [*F*(1, 19) = 16.9, *p* = 0.006], IL-9 [*F*(1, 19) = 13.0, *p* = 0.002], IL-12p70 [*F*(1, 19) = 15.1, *p* = 0.001], IL-17A [*F*(1, 19) = 4.748, *p* = 0.042], and IFN-γ [*F*(1, 19) = 6.2, *p* = 0.022].

Among anti-inflammatory cytokines (Tables 3 & 4, Fig. 5), morphine increased the concentration of IL-5 [*F*(1, 19) = 26.0, *p* < 0.001] and IL-13 [*F*(1, 19) = 39.6, *p* < 0.001] in the striatum, and IL-10 [F(1,19) = 7.4, p = 0.014] in the spinal cord, while Tat expression reduced protein levels of IL-10 in the striatum [*F*(1, 19) = 12.0, *p* = 0.003]. Morphine administration and Tat expression significantly interacted to reduce IL-4 [striatum, *F*(1,19) = 5.5, *p* = 0.030; spinal cord, *F*(1, 19) = 6.3, *p* = 0.022] expression within the striatum and spinal cord. In the striatum, morphine-treated Tat(+) mice had reduced IL-4 compared to saline-treated Tat(−) mice (*p* = 0.047) and morphine-treated Tat(−) mice (*p* = 0.008). In the spinal cord, morphine-treated Tat(−) mice showed significantly increased IL-4 compared to saline-treated controls (Tat(−), *p* = 0.007; Tat(+), *p* = 0.023) and morphine-treated, Tat(+) mice (*p* = 0.040).

Tat expression significantly reduced various chemokines and colony-stimulating factors (Tables 3 & 4, Fig. 6), within the striatum [G-CSF, *F*(1, 19) = 13.7, *p* = 0.002; GM-CSF, *F*(1, 19) = 7.700, *p* = 0.012; CXCL1, *F*(1, 19) = 15.3, *p* < 0.001; CCL2, *F*(1, 19) = 13.6, *p* = 0.002; CCL4, *F*(1, 19) = 18.4, *p* < 0.001; CCL5, *F*(1, 19) = 10.20, *p* = 0.005] and spinal cord [G-CSF, *F*(1, 19) = 6.1, *p* = 0.023; GM-CSF, *F*(1, 19 = 5.4, *p* = 0.032; CXCL1, *F*(1, 19) = 7.1, *p* = 0.015]. Morphine treatment increased chemokine concentrations within the spinal cord [G-CSF, *F*(1, 19) = 11.1, *p* = 0.004; GM-CSF, *F*(1, 19) = 11.3, *p* = 0.003; CCL2, *F*(1, 19) = 12.7, *p* = 0.002; CCL4, *F*(1, 19) = 20.3, *p* < 0.001; CCL5, *F*(1, 19) = 4.9, *p* = 0.039]. Morphine treatment increased striatal CCL5 [*F*(1, 19) = 10.2, *p* = 0.005]. No significant genotype by drug interactions were observed in either region. Overall, 3-month Tat exposure increased innate immune tolerance evidenced by reductions in specific cytokines (e.g., IL-1α, IL-12p40), while short-term morphine exposure acted as a secondary stressor revealing an allostatic shift in CNS baseline inflammatory responsiveness from sustained Tat exposure.

### Levels of endocannabinoids and related non-eCB lipids in the striatum and spinal cord

**Fig. 7:**
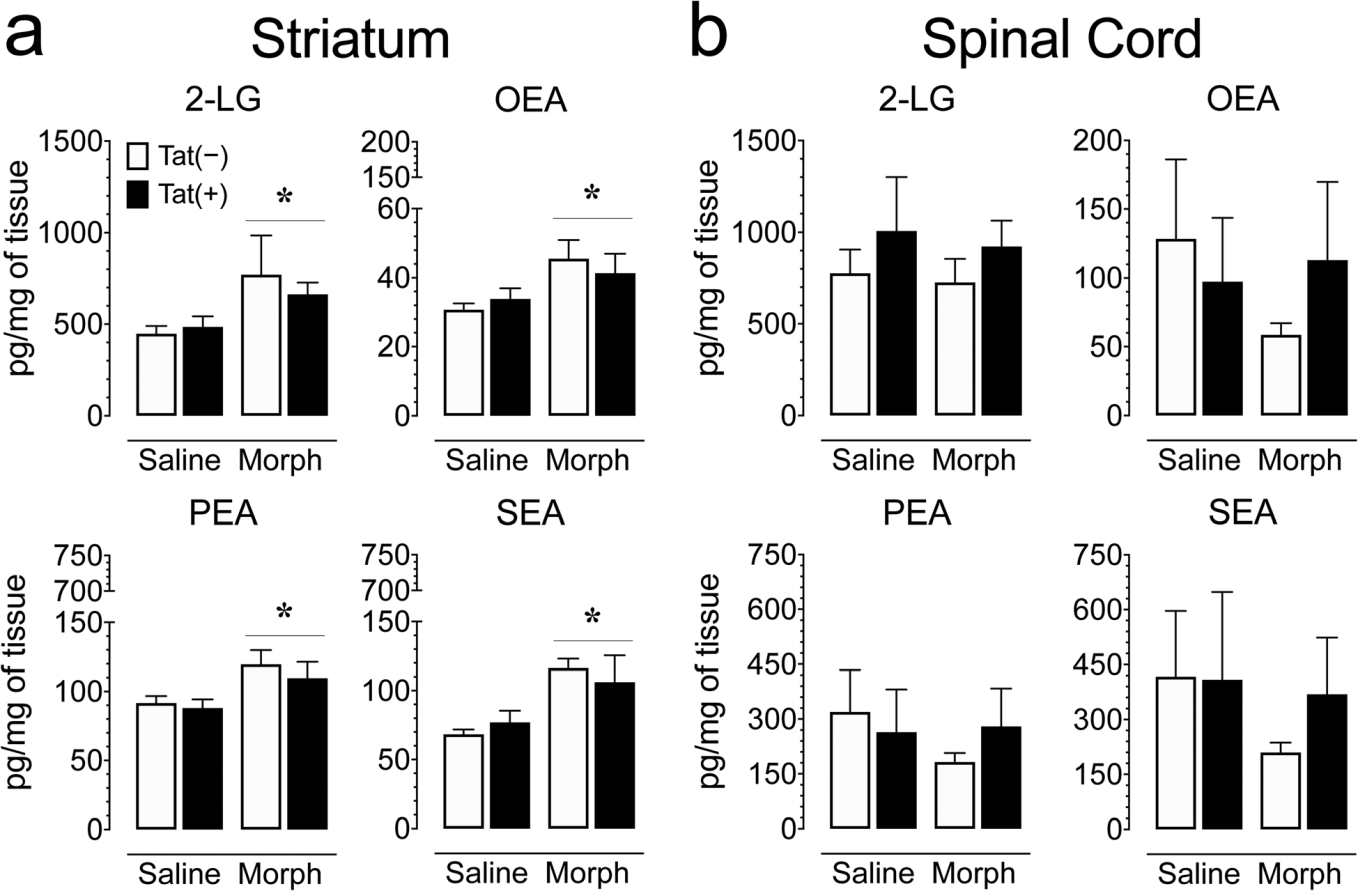
Morphine treatment increased 2-LG, OEA, PEA, and SEA concentrations (pg/mg) in the striatum but not spinal cord of Tat transgenic mice. Concentrations of 2-AG and AEA and other lipid molecules were assessed in the striatum and spinal cord on saline and morphine-treated Tat transgenic mice using LC/MS/MS. Lipid concentrations were normalized to pg/mg of tissue. **a** In the striatum, morphine significantly upregulated 2-LG, OEA, PEA, and SEA concentrations in Tat(+) and Tat(−) mice. **b** In the spinal cord, no significant effects were noted on any of the 4 measures. All data are expressed as mean ± SEM. Statistical significance was assessed by ANOVA followed by Tukey’s post hoc test; *p < 0.05 main effect of drug. *Morph* short-term (8-day) morphine injections. *n* = 3–5 mice per group

To assess the impact of chronic Tat and repeated morphine exposure on the endogenous cannabinoid system, changes in levels of endocannabinoids (eCBs) and other lipid signaling molecules were assessed in the striatum and spinal cord of Tat transgenic mice (*n* = 3–5 per group; Table 5, Fig. 7). CNS region effects were noted; 2-AG [*F*(1, 13) = 4.7, *p* = 0.050] and AEA [*F*(1, 13) = 7.3, *p* = 0.018] levels were reduced in the spinal cord compared to the striatum, whereas other lipids, including NAGly [*F*(1, 13) = 14.4, *p* = 0.002], 2-LG [*F*(1, 13) = 9.6, *p* = 0.008], OEA [*F*(1, 13) = 5.2, *p* = 0.040], PEA [*F*(1, 13) = 8.4, *p* = 0.013], DEA [*F*(1, 13) = 9.5, *p* = 0.009], and SEA [*F*(1, 13) = 7.9, *p* = 0.015], had higher levels in the spinal cord compared to the striatum. To assess Tat and morphine’s effect for each CNS region, separate two-way ANOVAs for the striatum and spinal cord were conducted with genotype and drug as between-subjects factors. No effects were noted for AEA and 2-AG on either of the two CNS structures and no effects were noted for any of the other lipids on the spinal cord (Table 5, Fig. 7b). For the striatum, no significant changes on any of these measures were noted for genotype, but morphine significantly upregulated four of the other lipids, including 2-LG [*F*(1, 13) = 7.6, *p* = 0.016], PEA [*F*(1, 13) = 7.4, *p* = 0.018], OEA [*F*(1, 13) = 6.6, *p* = 0.023], and SEA [*F*(1, 13) = 8.9, *p* = 0.011] (Fig. 7a). No differences were noted between individual groups. Thus, even though eCB levels were unchanged in either CNS structure, related non-eCB lipids, including 2-LG, OEA, PEA, and SEA, significantly increased with morphine exposure in the striatum but not in the spinal cord, irrespective of Tat exposure.

**Table 5:** Effects of Tat and morphine on levels of eCB and other lipid signaling molecules (pg/mg) in the striatum and spinal cord of Tat transgenic mice that were not significantly altered by Tat and/or morphine for any of the two CNS regions

CNS region	Lipids	Genotype	Repeated saline	Repeated morphine	Genotype effect		Drug effect		Genotype x drug	
	pg/mg		mean ± SEM	mean ± SEM	*F*_1, 13_	*p*	*F*_1, 13_	*p*	*F*_1, 13_	*p*
Striatum	2-AG	Tat(−)	11879.7 ± 1270.52	15441.7 ± 2440.16	< 1.0	0.93	2.1	0.17	< 1.0	0.52
		Tat(+)	13140.9 ± 2086.96	14476.7 ± 1229.29						
	AEA	Tat(−)	8.4 ± 0.52	9.1 ± 1.87	2.3	0.15	< 1.0	0.47	2.3	0.15
		Tat(+)	8.4 ± 1.08	6.4 ± 0.33						
	NAGly	Tat(−)	16.0 ± 1.18	17.0 ± 1.26	< 1.0	0.47	1.0	0.33	< 1.0	0.85
		Tat(+)	16.7 ± 1.55	18.10 ± 0.62						
	POEA	Tat(−)	4.9 ± 0.34	5.9 ± 0.93	1.1	0.31	< 1.0	0.36	1.5	0.24
		Tat(+)	5.0 ± 0.23	4.8 ± 0.39						
	LEA	Tat(−)	6.0 ± 0.74	7.9 ± 1.71	< 1.0	0.46	< 1.0	0.96	3.2	0.10
		Tat(+)	7.1 ± 1.33	5.2 ± 0.67						
	DEA	Tat(−)	2.8 ± 0.09	3.6 ± 0.91	< 1.0	0.39	1.1	0.32	1.7	0.22
		Tat(+)	2.9 ± 0.20	2.8 ± 0.16						
	DHEa	Tat(−)	16.1 ± 0.64	18.1 ± 4.25	< 1.0	0.66	< 1.0	0.58	2.9	0.11
		Tat(+)	18.4 ± 1.99	14.3 ± 0.61						
Spinal cord	2-AG	Tat(−)	11168.1 ± 1849.25	13770.4 ± 4148.01	1.4	0.26	1.4	0.26	< 1.0	0.90
		Tat(+)	9033.6 ± 993.83	11126.1 ± 1239.95						
	AEA	Tat(−)	5.8 ± 0.79	5.7 ± 2.03	< 1.0	0.77	< 1.0	0.60	< 1.0	0.55
		Tat(+)	5.3 ± 0.48	7.1 ± 1.98						
	NAGly	Tat(−)	22.8 ± 2.07	22.3 ± 4.38	< 1.0	0.43	< 1.0	0.74	< 1.0	0.56
		Tat(+)	19.6 ± 1.81	21.7 ± 1.19						
	POEA	Tat(−)	8.7 ± 3.12	4.9 ± 0.58	< 1.0	0.83	< 1.0	0.71	< 1.0	0.38
		Tat(+)	6.7 ± 2.02	8.3 ± 3.38						
	LEA	Tat(−)	10.6 ± 4.75	5.7 ± 1.40	< 1.0	0.66	< 1.0	0.78	< 1.0	0.52
		Tat(+)	9.5 ± 3.85	11.4 ± 6.26						
	DEA	Tat(−)	3.9 ± 0.30	3.8 ± 1.19	< 1.0	0.82	< 1.0	0.37	1.1	0.32
		Tat(+)	3.2 ± 0.42	4.3 ± 0.57						
	DHEa	Tat(−)	15.8 ± 0.55	18.4 ± 6.49	< 1.0	0.61	1.5	0.24	< 1.0	0.48
		Tat(+)	14.7 ± 1.02	24.9 ± 7.59						

Levels of eCB and non-eCB lipids in the striatum and spinal cord of repeated (8-day) saline- or morphine-treated Tat(−) and Tat(+) mice were not significantly affected by Tat and/or morphine for any of the two CNS regions. Data are expressed as mean ± SEM in pg/mg. Two-way ANOVAs for each CNS region were conducted with genotype and drug as between-subjects factors. *F* values and *p* values are presented from ANOVA results. *n* = 3–5 mice per group. All significant effects are represented in Fig. 7

### Levels of amino acids in the striatum and spinal cord

**Fig. 8:**
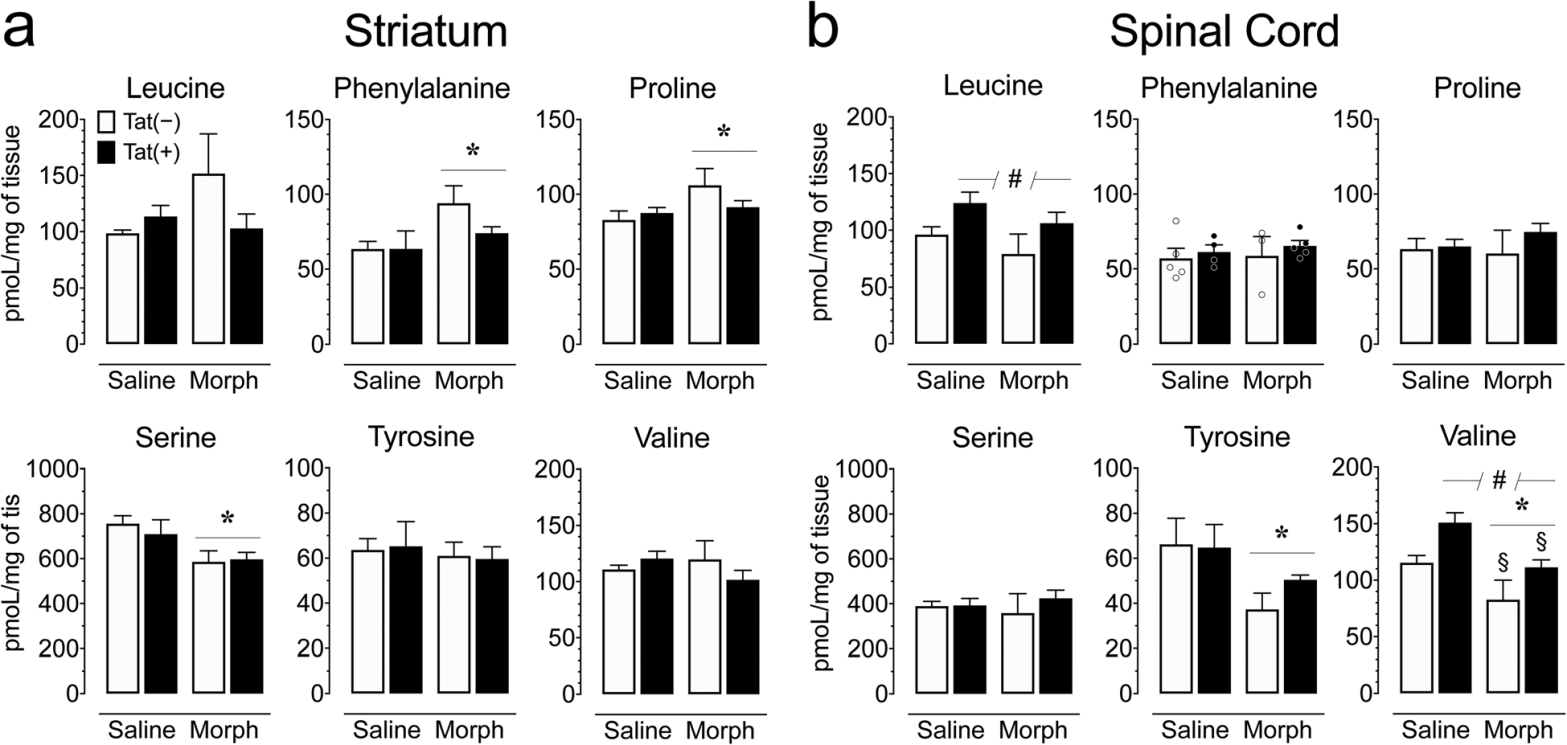
Amino acid levels (pmol/mg) are significantly altered by Tat exposure or morphine depending on the CNS region assessed. The concentrations of various amino acids were assessed in the striatum and spinal cord on saline and morphine-treated Tat transgenic mice using hydrophilic interaction chromatography (HILIC)-LC/MS/MS. Amino acid concentrations were normalized to pmol/mg of tissue. **a** In the striatum, morphine significantly upregulated phenylalanine and proline concentrations, and significantly downregulated serine levels in Tat(+) and Tat(−) mice. **b** In the spinal cord, Tat expression increased leucine and valine levels, whereas morphine significantly decreased valine and tyrosine levels. All data are expressed as mean ± SEM. Statistical significance was assessed by ANOVA followed by Tukey’s post hoc test; *p < 0.05 main effect of drug; ^#^*p* < 0.05 main effect of genotype; ^§^*p* < 0.05 vs. saline-exposed Tat(+) mice. *Morph* short-term (8-day) morphine injections. *n* = 3–5 mice per group

As neuroinflammation, assessed by Iba-1+ cells and 3-NT+ Iba-1+ double-labeled cells, was significantly upregulated in morphine-exposed Tat(+) mice, we determined the impact of chronic Tat and repeated morphine exposure on various amino acids in the striatum and spinal cord (*n* = 3–5 per group; Table 6, Fig. 8). Multiple amino acids including glutamic acid [*F*(1, 13) = 165.76, *p* < 0.008], glycine [*F*(1, 13) = 147.2, *p* < 0.001], phenylalanine [*F*(1, 13) = 5.0, *p* = 0.043], proline [*F*(1, 13) = 19.2, *p* = 0.001], and serine [*F*(1, 13) = 84.7, *p* < 0.001] were differentially expressed across CNS regions and indicated higher levels in the striatum compared to the spinal cord, except for glycine. To assess Tat and morphine’s effect for each CNS region, separate two-way ANOVAs for the striatum and spinal cord were conducted with genotype and drug as between-subjects factors. No effects were noted for arginine, aspartic acid, glutamic acid, and glycine on any of the two CNS structures (Table 6). For the striatum, no significant changes on any of the other measures were noted for Tat expression, but morphine significantly upregulated phenylalanine [*F*(1, 13) = 6.5, *p* = 0.024] and proline [*F*(1, 13) = 4.7, *p* = 0.050], and significantly downregulated serine [*F*(1, 13) = 9.9, *p* = 0.008] (Fig. 8a). No differences were noted between individual groups. For the spinal cord, Tat expression significantly upregulated leucine [*F*(1, 13) = 7.0, *p* = 0.020] and valine [*F*(1, 13) = 12.5, *p* = 0.004]. Further, morphine downregulated tyrosine and valine [*F*(1, 13) = 5.5, *p* = 0.035 and *F*(1, 13) = 15.7, *p* = 0.002, respectively] (Fig. 8b). For valine, saline-treated Tat(+) mice demonstrated increased levels compared to morphine-treated Tat(−) mice (*p* = 0.002) and morphine-treated Tat(+) mice (*p* = 0.041; Fig. 8b). Together, these data show that the amino acids phenylalanine and proline were significantly increased by repeated morphine exposure in the striatum, whereas in the spinal cord, Tat exposure increased amino acids leucine and valine, while morphine decreased levels of tyrosine and valine.

**Table 6:** Effects of Tat and morphine on amino acid levels (pg/mg) in the striatum and spinal cord of Tat transgenic mice that were not significantly altered by Tat and/or morphine for any of the two CNS regions

CNS Region	Lipids	Geno-type	Repeated Saline	Repeated Morphine	Genotype Effect		Drug Effect		Genotype x Drug	
	pg/mg		mean ± SEM	mean ± SEM	***F***_***1, 13***_	***p***	***F***_***1, 13***_	***p***	***F***_***1, 13***_	***p***
**Striatum**	**Arginine**	Tat(−)	68.5 ± 4.98	78.5 ± 8.12	< 1.0	0.81	3.3	0.09	< 1.0	0.81
		Tat(+)	68.5 ± 4.26	76.1 ± 2.85						
	**Aspartic Acid**	Tat(−)	2937.1 ± 147.50	2943.6 ± 323.69	< 1.0	0.83	< 1.0	0.80	< 1.0	0.78
		Tat(+)	3049.2 ± 240.15	2927.9 ± 207.52						
	**Glutamic Acid**	Tat(−)	9774.8 ± 373.58	9707.57 ± 964.41	< 1.0	0.80	< 1.0	0.66	< 1.0	0.58
		Tat(+)	9605.3 ± 737.84	10,171.0 ± 259.75						
	**Glycine**	Tat(−)	1276.5 ± 120.56	1375.7 ± 150.47	< 1.0	0.59	< 1.0	0.63	< 1.0	0.79
		Tat(+)	1240.2 ± 162.27	1269.0 ± 81.91						
**Spinal Cord**	**Arginine**	Tat(−)	60.4 ± 4.37	66.7 ± 16.23	1.5	0.24	2.9	0.11	< 1.0	0.40
		Tat(+)	63.1 ± 1.99	82.3 ± 6.92						
	**Aspartic Acid**	Tat(−)	3237.1 ± 134.52	3031.0 ± 742.06	< 1.0	0.64	< 1.0	0.90	< 1.0	0.47
		Tat(+)	3148.3 ± 241.28	3442.9 ± 293.20						
	**Glutamic Acid**	Tat(−)	6286.3 ± 403.32	5867.4 ± 1186.47	< 1.0	0.99	< 1.0	0.65	< 1.0	0.77
		Tat(+)	6128.7 ± 252.56	6031.6 ± 418.03						
	**Glycine**	Tat(−)	4221.2 ± 165.88	3868.8 ± 780.64	< 1.0	0.97	< 1.0	0.63	1.8	0.20
		Tat(+)	3647.5 ± 367.56	4414.7 ± 405.38						

Levels of amino acids in the striatum and spinal cord of repeated (8-day) saline- or morphine-treated Tat(−) and Tat(+) mice were not significantly affected by Tat and/or morphine for any of the two CNS regions. Data are expressed as mean ± SEM in pg/mg. Two-way ANOVAs for each CNS region were conducted with genotype and drug as between-subjects factors. *F* values and *p* values are presented from ANOVA results. *n* = 3–5 mice per group. All significant effects are represented in Fig. 8

## Discussion

The present study investigated short-term (8-day) morphine’s effects in the Tat transgenic mouse model on antinociceptive tolerance, brain inflammatory processes, and expression levels of eCB, related non-eCB lipids and amino acids. Here, we report that Tat expression enhanced the magnitude of morphine tolerance specifically for the tail-flick task with less robust changes in the supraspinal-related hot-plate assay, potentially due to the small sample size that increased the likelihood of type II error. It also should be noted that the data for the morphine-exposed Tat(+) mice are based on estimated ED_50_ value and therefore caution needs to be exercised when interpreting the data. Additionally, Tat expression and morphine exposure significantly decreased body mass, which has been demonstrated in previous studies [98, 101] and is a well-established effect of administering high dose, acute morphine. Interestingly, a sustained (3-month) Tat insult appeared to induce innate immune tolerance because Tat exposure alone (1) no longer significantly increased microglial reactivity, (2) failed to increase the brain inflammatory marker [^18^F]-PBR111 during PET imaging, and (3) reduced levels of specific cytokines (e.g., IL-1α, IL-12p40), which are elevated by shorter duration (28-day) Tat induction [54]. Furthermore, short-term, escalating morphine exposure (designed to mimic accelerated abuse of opiates as seen with prescription opiate abuse) revealed that prolonged Tat exposure caused an allostatic shift in baseline inflammatory processes in the CNS. Moreover, whereas no changes in eCB levels were noted in either of the two CNS regions, other related non-eCB lipids, including 2-LG, OEA, PEA, and SEA significantly increased with repeated short-term morphine exposure in the striatum but not in the spinal cord, irrespective of Tat exposure. Absolute levels of these lipids were greater in the spinal cord than striatum, collectively suggesting fundamental differences in lipid biosynthesis/degradation and responsiveness to opiates between the two regions.

### Amino acids

Besides a suggested role of glial activation in morphine-induced antinociceptive tolerance, Tat exposure induced specific alterations in various amino acids in a CNS region-dependent manner. Tat elicited an upregulation of the branched-chain (having an aliphatic side chain) amino acids (BCAAs) leucine and valine in the spinal cord (isoleucine as the third BCAA was not assessed in this study), which participate directly and indirectly in intermediary metabolism, protein synthesis, and in neuropeptide neurotransmitters biosynthesis [102–104]. Whereas anti-inflammatory activities have been related to BCAAs [74, 105], high brain concentrations of BCAA may have detrimental effects on protein synthesis, neurotransmitter synthesis and release, and promote oxidative stress and inflammation [76, 106]. As inflammation and oxidative stress have been demonstrated after Tat exposure in vivo and in vitro [34, 37, 38, 54, 107–109], understanding the role of BCAAs in Tat toxicity requires more detailed investigation.

Short-term morphine exposure significantly changed levels of the aromatic amino acids (i.e., having an aromatic side chain) phenylalanine and tyrosine in a CNS region-dependent manner. These findings are interesting considering the essential role of phenylalanine and its conversion to tyrosine in catecholamine synthesis [110] and bearing in mind the wide-spread alterations in catecholamines (i.e., dopamine, norepinephrine, and epinephrine) in neuroHIV [111]. Specifically, it has been shown that immune activation can lead to diminished conversion of phenylalanine to tyrosine as evidenced by a higher phenylalanine/tyrosine (P/T) ratio [112], which has been shown to be present in HIV-infected patients [113] and potentially contributes to the dopamine deficiency in people living with HIV [114]. The downregulation of tyrosine in the spinal cord (but not striatum) following repeated morphine treatment supports findings in brain tissue of mice chronically exposed to heroin [60] and has been shown to allay some of the effects of opioid withdrawal [73]. In turn, we also report here that morphine leads to a significant upregulation of phenylalanine in the striatum (but not spinal cord) of Tat(−) mice. Similarly, rats self-administrating heroin [59] and mice undergoing heroin withdrawal [60] showed elevated levels of phenylalanine in serum. The lack of elevated phenylalanine concentrations in the spinal cord suggests regional differences in its metabolism (similar to tyrosine), which may coincide with regional differences in morphine concentration in the CNS [40]. Interestingly, Tat induction appeared to negate morphine-dependent increases in striatal phenylalanine in our study and Tat and/or HIV exposure have been associated with pathophysiological alterations in dopamine [115–118].

Lastly, proline and phenylalanine have been reported to display anti-inflammatory effects [74, 119] and were significantly upregulated in the striatum by short-term, escalating morphine exposure. The anti-inflammatory effects of morphine have not been clearly demonstrated but various recent studies point toward that direction [54, 120]. Additionally, in the present study, we show that morphine administration significantly downregulates the amino acid serine in the striatum. Though serine’s effects on the CNS are currently debated [121–123], the downregulation of serine by morphine point again to a morphine-induced anti-inflammatory effect [121], which is also supported by our findings of morphine-induced increases in the anti-inflammatory cytokines IL-4 and IL-10 in the spinal cord.

### Endocannabinoids

The upregulation of anti-inflammatory amino acids by short-term morphine exposure in the striatum could be potentially explained by the changes observed on eCBs and related non-eCB lipid signaling molecules. Even though no effects were noted for AEA and 2-AG, morphine exposure significantly upregulated other lipids in the striatum, which are structurally related to AEA (PEA, OEA, SEA) and 2-AG (2-LG), but do not activate cannabinoid receptors. PEA and OEA activate other G-protein-coupled receptors, including GPR55 and GPR119 [124, 125], and other receptor systems including peroxisome proliferator-activated alpha receptors (PPAR-α [126, 127];), and/or transient receptor potential vanilloid 1 (TRPV1) channels [128–130]. Notably, PEA, OEA, and SEA share the same hydrolytic enzyme as AEA, fatty acid amide hydrolase (FAAH). The average concentrations of the lipids are approximately or above 100 pmol/g tissue, whereas AEA is found at much lower levels of 1**–**30 pmol/g [131, 132]. When discussing the biological roles of PEA, OEA, and SEA, it can often be difficult to differentiate involvement of these lipids with those of AEA since their levels in the tissues are often altered in parallel with changes in AEA [133–136]. However, some studies indicate that PEA, OEA, and/or SEA may have individual non-cannabinoid roles, e.g., OEA and PEA exert anti-inflammatory and antioxidant neuroprotection via activation of PPAR-α [126, 137–139]. The targets for SEA are less clear, even though it is known that it lacks affinity for the CB_1_R, but it does have some cannabimimetic actions in rats in vivo [140]. Further, PPARs can reduce the development of morphine tolerance by activating PPAR-γ [65]. We speculate that the morphine-dependent increases in production of PEA, OEA, SEA, and 2-LG, as well as the upsurges in anti-inflammatory (phenylalanine and proline) and decreases in proinflammatory (serine) amino acids, represent a homeostatic response to the rapid increases in neuroinflammation seen with acute morphine exposure in the presence of an inflammatory insult such as Tat. The activation of selective PPARs, TRPV1, GPR55, and/or GPR119 by these endogenous ligands may be important in breaking the cycle and return to steady state, and one or more of these receptors may be important therapeutic targets for opiate abuse and HIV-1 comorbidity or for the management of pain.

### Neuroinflammation

Several studies demonstrate increased gliosis, macrophage turnover, and encephalitis in HIV-1 infected humans who abuse opiates [10–12], which also has been supported in various mouse models of neuroHIV-opiate co-exposure [141–143]. In the current study, combined Tat and morphine together induced significant increases in the number of microglia and the proportion displaying nitrosative products in the striatum and spinal cord, which were not evident with Tat alone. Additionally, abnormal microglial morphology was assessed in the spinal cord upon Tat and morphine exposure. By contrast, increases in TSPO levels via PET imaging were not demonstrated through the [^18^F]-PRB111 ligand. The discrepancy between the TSPO and Iba-1 results could be that the [^18^F]-PRB111 ligand lacks sufficient sensitivity to detect small changes in TSPO levels or subtle changes in neuroinflammation. Although increased [^18^F]-PRB111 uptake has been demonstrated in several animal models of neurological diseases including epilepsy [144, 145], multiple sclerosis [146], and schizophrenia [147], these models display extensive inflammation and postmortem pathology [144–147], whereas abnormalities in the HIV-1 Tat transgenic mouse model are more subtle [148] with opioid exposure inducing modest gliosis [29, 149]. Alternatively, increases in TSPO may be most dramatic in proinflammatory “M1” microglia when initially activated and may decline with sustained activation or upon switching to a repair/remodeling “M2” phenotype [150–152]—although there is clearly a spectrum of functional states beyond binary M1/M2 polarization [153]. With sustained Tat exposure, we speculate that microglia shift toward a less proinflammatory, M2-like phenotype and that innate immune responsiveness in the CNS becomes increasingly tolerant to Tat. In fact, this assertion is supported by the reductions seen in key regulatory cytokines and chemokines, increases in anti-inflammatory cytokines (discussed below), and the absence of Tat-dependent increases in microglial numbers and/or activation as assessed by immunohistochemistry and PET imaging.

A previous study has demonstrated increased binding of the related TPSO PET imaging [^11^C]*N*,*N*-diethyl-2-[2-(4-methoxyphenyl)-5,7-dimethylpyrazolo[1,5-a]pyrimidin-3-yl]acetamide ([^11^C]-DPA-713) ligand in the brains of HIV-1 infected patients [154]. Although the reason for the disparity is uncertain, unlike Tat in which expression levels are designed to remain constant with continuous DOX treatment, neuroHIV can persist as a dynamic, smoldering infection (albeit at low levels) despite cART and may be more likely to promote a proinflammatory phenotype than constant Tat exposure. Although the TSPO PET imaging and Iba-1 estimates of microglial density/reactivity were in general agreement following sustained Tat exposure, the measures did not agree with combined morphine and Tat exposure. While the reason(s) for the disparity is (are) uncertain, short-term morphine exposure may revert “M2” microglia toward an “M1” proinflammatory phenotype as evidenced by increases in some proinflammatory cytokines only seen with Tat and morphine co-exposure (discussed below). Alternatively, the discrepancy between the TSPO PET imaging and Iba-1 findings may also relate to the selectivity of [^18^F]-PRB111 as a microglial marker. While Iba-1 is selective for microglia and macrophages, the specific glial type(s) upregulating TPSO during inflammation [155] remain(s) a point of discussion as TPSO activation has been demonstrated in astrocytes [156–158].

Repeated inflammatory insults can induce long-term innate immune tolerance within resident microglia in the CNS [159–161]. A recent study demonstrated that a single intraperitoneal (i.p.) injection of lipopolysaccharide (LPS) potentiated microglial inflammatory activity in response to a secondary insult (transgenic amyloid precursor protein expression) 6 months later, whereas repeated i.p. doses of LPS dampened IL-6 production, with similar trends in IL-1β, IL-10 and CXCL1 [57], thus indicating longer-term immune tolerance akin to the immunosuppression [55–57]. Multiple LPS injections also dampened acute IL-1β production in a brain ischemia model, which correlated to reduced microglial activation and improved neuronal survival [57]. Reduced microglial IL-1β production was observed up to 32 weeks after mice were primed with a peripheral LPS injection [160]. In several of these studies, innate immune tolerance involved epigenetic reprogramming dependent on histone deacetylasese-1 and -2 as well as TAK1*-*dependent NF-κB signaling [57, 160], though additional mechanisms are likely to be operative.

Sustained (3-month) Tat exposure not only failed to increase but actually reduced levels of many cytokines and chemokines. This included G-CSF, GM-CSF, IL-5, and CXCL1 in the spinal cord, and notably IL-1α, IL-17A, IL-10, G-CSF, GM-CSF, CXCL1, CCL2, CCL4, and CCL5 in the striatum, which differs from the cytokine increases seen with ≤ 1-month Tat exposure durations [38, 54]. Microgliosis was also not evident in the striatum or spinal cord, despite being present in the striatum following 48 h [29] or 12 days [31] exposure to Tat alone. We speculate that the inflammatory contributions of microglia per se differ in each region and situation, and underlie unique differences in the profile of inflammatory factors (including cytokines and lipid mediators) that drive regional and temporal differences in the pathology. Single-cell RNA-sequencing and related strategies would be advantageous in confirming the role of microglial heterogeneity as underlying spatiotemporal differences in opioid-HIV comorbid pathobiology. Collectively, our findings suggest that, despite some differences in the profile of cytokine changes between the striatum and spinal cord, the innate immune response had become tolerant to Tat. Moreover, the normally coordinated production of pro- and anti-inflammatory cytokines also became unbalanced, indicating that Tat-induced innate immune tolerance in the CNS is accompanied by immune dysregulation, which is emblematic in HIV-infected individuals [162] and frequently seen in neuroHIV [163–165].

## Conclusions

In the present study, we evaluated the impact of a short-term ramping morphine regimen sufficient to cause opiate tolerance to mimic the escalating consumption seen with prescription opiate abuse in mutant mouse model displaying chronic elevations of Tat (Fig. 2). Although 2–14 days of morphine and Tat/HIV co-exposure typically exacerbates cytokine production, gliosis, and bystander neuronal injury in vitro [30, 37, 43, 166] and in vivo [29, 38], 3 months of Tat induction elicited a far less fulminant morphine-dependent interactive pathology. Morphine and Tat co-exposure elevated 3-NT in microglia, but failed to increase microglial numbers as seen previously following 48 h or 12-day Tat induction [29, 31]. Importantly, the final 80 mg/kg s.c. morphine dose used in prior studies delivers approximately the same amount of morphine during 24 h as a 25 mg time-release pelleted implant (NIDA Drug Supply System) [29, 35, 38]. Thus, the Tat-induced reductions in certain cytokines (e.g., IL-1α, IL-17A) suggests that adaptive responses of some cytokines to Tat are more coordinated across some brain regions than others. Importantly, by delaying exposure until these adaptative processes had occurred, the response to morphine differed markedly from that previously seen with a shorter duration of Tat exposure. Accordingly, altering the timing of morphine co-exposure revealed an allostatic shift in CNS baseline cytokine responsiveness resulting from sustained Tat exposure. The manner by which duration of HIV infection might differentially interact with opiate exposure to affect other outcome measures, especially neuronal injury and dysfunction, remains uncertain but is a compelling area for further investigation. In sum, our results suggest that the nature and timing of opiate and HIV comorbid interactions have dramatically different consequences for addictive behavior and neuroHIV pathogenesis.

## Data Availability

The dataset used and/or analyzed during the current study are available from the corresponding author on reasonable request.

## Abbreviations

2-AG: 2-Arachidonoyl glycerol
2-LG: 2-Linoleoyl glycerol
3-NT: 3-Nitrotyrosine
[^11^C]-DPA-713: [^11^C]*N*,*N*-Diethyl-2-[2-(4-methoxyphenyl)-5,7-dimethylpyrazolo[1,5-a]pyrimidin-3-yl]acetamide
[^18^F]-PBR111: 2-(6-Chloro-2-(4-(3-^18^F-fluoropropoxy)phenyl)imidazo[1,2-a]pyri-din-3-yl)-*N*,*N*-diethylacetamide
AEA: Anandamide/*N*-arachidonoyl ethanolamine
ANOVA: Analysis of variance
BBB: Blood-brain barrier
BCAAs: Branched-chain amino acids
BRIC: Biomedical Research Imaging Center
cART: Combined antiretroviral therapy
CB_1_R: Cannabinoid receptor type 1
CCLx: C-C chemokine ligand type x
CI: Confidence interval
CNS: Central nervous system
CT: Computed tomography
CXCL1: C-X-C motif chemokine ligand type 1
DEA: Docosatetraenyl ethanolamide
DHEa: Docosahexaenoyl ethanolamide
DOR: δ-opioid receptor
DOX: Doxycycline
eCB: Endocannabinoid
ED_50_: 50% effective dose
FAAH: Fatty acid amide hydrolase
GFAP: Glial fibrillary acidic protein
GPR119: G-protein-coupled receptor 119
GPR55: G-protein-coupled receptor 55
G-CSF: Granulocyte-colony stimulating factor
GM-CSF: Granulocyte-macrophage colony-stimulating factor
HIV-1: Human immunodeficiency virus type 1
HILIC: Hydrophilic interaction chromatography
HPLC: High-performance liquid chromatography
IACUC: Institutional Animal Care and Use Committee
Iba-1: Ionized calcium binding adaptor molecule 1
IFN-γ: Interferon gamma
IL-x: Interleukin x
IP: Immunoprecipitation
i.p.: Intraperitoneal
LEA: Linoleoyl ethanolamide
LS/MS/MS: Liquid chromatography coupled with tandem mass spectrometry
LPS: Lipopolysaccharide
MOR: μ-Opioid receptor
MRI: Magnetic resonance imaging
NAGly: *N*-arachidonoyl glycine
NIH: National Institutes of Health
NMDAR: *N*-methyl-d-aspartate receptor
OEA: *N*-oleoyl ethanolamide
PBS: Phosphate-buffered saline
PEA: *N*-palmitoyl ethanolamide
PET: Positron emission tomography
PKU: Phenylketonuria
POEA: *N*-palmitoleoyl ethanolamide
PPAR: Peroxisome proliferator activator receptor
rtTA: Reverse tetracycline transactivator
SEA: *N*-stearoyl ethanolamine
SEM: Standard error of the mean
s.c.: Subcutaneous
SUV: Standardized uptake value
Tat: Transactivator of transcription
TNF-α: Tumor necrosis factor alpha
TRE: Tetracycline responsive element
TRP: Transient receptor potential cation channel subfamily V member 1
TSPO: 18-kDa translocator protein

## References

[1] Meng J, Sindberg GM, Roy S (2015). Disruption of gut homeostasis by opioids accelerates HIV disease progression. Front Microbiol.

[2] Wang X, Tan N, Douglas SD, Zhang T, Wang YJ, Ho WZ (2005). Morphine inhibits CD8+ T cell-mediated, noncytolytic, anti-HIV activity in latently infected immune cells. J Leukoc Biol.

[3] Edelman EJ, Cheng DM, Krupitsky EM, Bridden C, Quinn E, Walley AY, Lioznov DA, Blokhina E, Zvartau E, Samet JH (2015). Heroin use and HIV disease progression: results from a pilot study of a Russian cohort. AIDS Behav.

[4] Weisberg DF, Gordon KS, Barry DT, Becker WC, Crystal S, Edelman EJ, Gaither J, Gordon AJ, Goulet J, Kerns RD (2015). Long-term prescription of opioids and/or benzodiazepines and mortality among HIV-infected and uninfected patients. J Acquir Immune Defic Syndr.

[5] Smith HS (2011). Treatment considerations in painful HIV-related neuropathy. Pain Physician.

[6] Onen NF, Barrette EP, Shacham E, Taniguchi T, Donovan M, Overton ET (2012). A review of opioid prescribing practices and associations with repeat opioid prescriptions in a contemporary outpatient HIV clinic. Pain Pract.

[7] Liu B, Liu X, Tang SJ (2016). Interactions of opioids and HIV infection in the pathogenesis of chronic pain. Front Microbiol.

[8] Krashin DL, Merrill JO, Trescot AM (2012). Opioids in the management of HIV-related pain. Pain Physician.

[9] Bell JE, Brettle RP, Chiswick A, Simmonds P (1998). HIV encephalitis, proviral load and dementia in drug users and homosexuals with AIDS. Effect of neocortical involvement. Brain.

[10] Bell JE, Arango JC, Anthony IC (2006). Neurobiology of multiple insults: HIV-1-associated brain disorders in those who use illicit drugs. J Neuroimmune Pharmacol.

[11] Anthony IC, Arango JC, Stephens B, Simmonds P, Bell JE (2008). The effects of illicit drugs on the HIV infected brain. Front Biosci.

[12] Anthony IC, Ramage SN, Carnie FW, Simmonds P, Bell JE (2005). Does drug abuse alter microglial phenotype and cell turnover in the context of advancing HIV infection?. Neuropathol Appl Neurobiol.

[13] Dutta R, Roy S (2012). Mechanism(s) involved in opioid drug abuse modulation of HAND. Curr HIV Res.

[14] Saylor D, Dickens AM, Sacktor N, Haughey N, Slusher B, Pletnikov M, Mankowski JL, Brown A, Volsky DJ, McArthur JC (2016). HIV-associated neurocognitive disorder - pathogenesis and prospects for treatment. Nat Rev Neurol.

[15] Hauser KF, Fitting S, Dever SM, Podhaizer EM, Knapp PE (2012). Opiate drug use and the pathophysiology of neuroAIDS. Curr HIV Res.

[16] Hauser KF, Knapp PE (2014). Interactions of HIV and drugs of abuse: the importance of glia, neural progenitors, and host genetic factors. Int Rev Neurobiol.

[17] Tavazzi E, Morrison D, Sullivan P, Morgello S, Fischer T (2014). Brain inflammation is a common feature of HIV-infected patients without HIV encephalitis or productive brain infection. Curr HIV Res.

[18] Slim J, Saling CF (2016). A review of management of inflammation in the HIV population. Biomed Res Int.

[19] Bell JE (2004). An update on the neuropathology of HIV in the HAART era. Histopathology.

[20] Gray F, Chretien F, Vallat-Decouvelaere AV, Scaravilli F (2003). The changing pattern of HIV neuropathology in the HAART era. J Neuropathol Exp Neurol.

[21] Gras G, Chretien F, Vallat-Decouvelaere AV, Le Pavec G, Porcheray F, Bossuet C, Leone C, Mialocq P, Dereuddre-Bosquet N, Clayette P (2003). Regulated expression of sodium-dependent glutamate transporters and synthetase: a neuroprotective role for activated microglia and macrophages in HIV infection?. Brain Pathol.

[22] Anthony IC, Ramage SN, Carnie FW, Simmonds P, Bell JE (2005). Influence of HAART on HIV-related CNS disease and neuroinflammation. J Neuropathol Exp Neurol.

[23] Gannon P, Khan MZ, Kolson DL (2011). Current understanding of HIV-associated neurocognitive disorders pathogenesis. Curr Opin Neurol.

[24] Persidsky Y, Ho W, Ramirez SH, Potula R, Abood ME, Unterwald E, Tuma R (2011). HIV-1 infection and alcohol abuse: neurocognitive impairment, mechanisms of neurodegeneration and therapeutic interventions. Brain Behav Immun.

[25] Sorrell ME, Hauser KF (2014). Ligand-gated purinergic receptors regulate HIV-1 Tat and morphine related neurotoxicity in primary mouse striatal neuron-glia co-cultures. J Neuroimmune Pharmacol.

[26] Bokhari SM, Yao H, Bethel-Brown C, Fuwang P, Williams R, Dhillon NK, Hegde R, Kumar A, Buch SJ (2009). Morphine enhances Tat-induced activation in murine microglia. J Neurovirol.

[27] Gupta S, Knight AG, Gupta S, Knapp PE, Hauser KF, Keller JN, Bruce-Keller AJ (2010). HIV-Tat elicits microglial glutamate release: role of NAPDH oxidase and the cystine-glutamate antiporter. Neurosci Lett.

[28] Hahn YK, Podhaizer EM, Farris SP, Miles MF, Hauser KF, Knapp PE (2015). Effects of chronic HIV-1 Tat exposure in the CNS: heightened vulnerability of males versus females to changes in cell numbers, synaptic integrity, and behavior. Brain Struct Funct.

[29] Bruce-Keller AJ, Turchan-Cholewo J, Smart EJ, Geurin T, Chauhan A, Reid R, Xu R, Nath A, Knapp PE, Hauser KF (2008). Morphine causes rapid increases in glial activation and neuronal injury in the striatum of inducible HIV-1 Tat transgenic mice. Glia.

[30] El-Hage N, Bruce-Keller AJ, Yakovleva T, Bazov I, Bakalkin G, Knapp PE, Hauser KF (2008). Morphine exacerbates HIV-1 Tat-induced cytokine production in astrocytes through convergent effects on [Ca^2+^]_i_. NF-κB trafficking and transcription. PLoS One.

[31] Zou S, Fitting S, Hahn YK, Welch SP, El-Hage N, Hauser KF, Knapp PE (2011). Morphine potentiates neurodegenerative effects of HIV-1 Tat through actions at μ-opioid receptor-expressing glia. Brain.

[32] Nookala AR, Kumar A (2014). Molecular mechanisms involved in HIV-1 Tat-mediated induction of IL-6 and IL-8 in astrocytes. J Neuroinflammation.

[33] Turchan-Cholewo J, Dimayuga FO, Gupta S, Keller JN, Knapp PE, Hauser KF, Bruce-Keller AJ (2009). Morphine and HIV-Tat increase microglial-free radical production and oxidative stress: possible role in cytokine regulation. J Neurochem.

[34] Fitting S, Zou S, El-Hage N, Suzuki M, Paris JJ, Schier CJ, Rodriguez JW, Rodriguez M, Knapp PE, Hauser KF. Opiate addiction therapies and HIV-1 Tat: interactive effects on glial [Ca^2+^]_i_, oxyradical and neuroinflammatory chemokine production and correlative neurotoxicity. Curr HIV Res. 2014;12:424–34. 10.2174/1570162X1206150311161147 PMC4475822 25760046

[35] El-Hage N, Wu G, Wang J, Ambati J, Knapp PE, Reed JL, Bruce-Keller AJ, Hauser KF (2006). HIV-1 Tat and opiate-induced changes in astrocytes promote chemotaxis of microglia through the expression of MCP-1 and alternative chemokines. Glia.

[36] El-Hage N, Bruce-Keller AJ, Knapp PE, Hauser KF (2008). CCL5/RANTES gene deletion attenuates opioid-induced increases in glial CCL2/MCP-1 immunoreactivity and activation in HIV-1 Tat-exposed mice. J Neuroimmune Pharmacol.

[37] El-Hage N, Gurwell JA, Singh IN, Knapp PE, Nath A, Hauser KF (2005). Synergistic increases in intracellular Ca^2+^, and the release of MCP-1, RANTES, and IL-6 by astrocytes treated with opiates and HIV-1 Tat. Glia.

[38] Fitting S, Xu R, Bull C, Buch SK, El-Hage N, Nath A, Knapp PE, Hauser KF (2010). Interactive comorbidity between opioid drug abuse and HIV-1 Tat: chronic exposure augments spine loss and sublethal dendritic pathology in striatal neurons. Am J Pathol.

[39] Mahajan SD, Aalinkeel R, Sykes DE, Reynolds JL, Bindukumar B, Fernandez SF, Chawda R, Shanahan TC, Schwartz SA (2008). Tight junction regulation by morphine and HIV-1 tat modulates blood-brain barrier permeability. J Clin Immunol.

[40] Leibrand CR, Paris JJ, Jones AM, Masuda QN, Halquist MS, Kim WK, Knapp PE, Kashuba ADM, Hauser KF, McRae M (2019). HIV-1 Tat and opioids act independently to limit antiretroviral brain concentrations and reduce blood-brain barrier integrity. J Neurovirol.

[41] Dalvi P, Sharma H, Chinnappan M, Sanderson M, Allen J, Zeng R, Choi A, O'Brien-Ladner A, Dhillon NK (2016). Enhanced autophagy in pulmonary endothelial cells on exposure to HIV-Tat and morphine: role in HIV-related pulmonary arterial hypertension. Autophagy.

[42] Malik S, Khalique H, Buch S, Seth P (2011). A growth factor attenuates HIV-1 Tat and morphine induced damage to human neurons: implication in HIV/AIDS-drug abuse cases. PLoS One.

[43] Fitting S, Knapp PE, Zou S, Marks WD, Bowers MS, Akbarali HI, Hauser KF. Interactive HIV-1 Tat and morphine-induced synaptodendritic injury is triggered through focal disruptions in Na^+^ influx, mitochondrial instability, and Ca^2+^ overload. J Neurosci. 2014;34:12850–64. 10.1523/JNEUROSCI.5351-13.2014 PMC4166164 25232120

[44] Gurwell JA, Nath A, Sun Q, Zhang J, Martin KM, Chen Y, Hauser KF (2001). Synergistic neurotoxicity of opioids and human immunodeficiency virus-1 Tat protein in striatal neurons in vitro. Neuroscience.

[45] Turchan-Cholewo J, Dimayuga FO, Ding Q, Keller JN, Hauser KF, Knapp PE, Bruce-Keller AJ (2008). Cell-specific actions of HIV-Tat and morphine on opioid receptor expression in glia. J Neurosci Res.

[46] Regan PM, Langford D, Khalili K (2016). Regulation and functional implications of opioid receptor splicing in opioid pharmacology and HIV pathogenesis. J Cell Physiol.

[47] Dever SM, Costin BN, Xu R, El-Hage N, Balinang J, Samoshkin A, O'Brien MA, McRae M, Diatchenko L, Knapp PE, Hauser KF. Differential expression of the alternatively spliced *OPRM1* isoform μ-opioid receptor-1K in HIV-infected individuals. AIDS. 2014;28:19–30. 10.1097/QAD.0000000000000113 PMC3939043 24413261

[48] Dever SM, Xu R, Fitting S, Knapp PE, Hauser KF (2012). Differential expression and HIV-1 regulation of μ-opioid receptor splice variants across human central nervous system cell types. J Neurovirol.

[49] Kraus J: Regulation of μ-opioid receptors by cytokines. Front Biosci (Schol Ed). 2009;1:164–70. 10.2741/s16 19482692

[50] Kraus J, Borner C, Giannini E, Hollt V. The role of nuclear factor κB in tumor necrosis factor-regulated transcription of the human μ-opioid receptor gene. Mol Pharmacol. 2003;64:876–84. 10.1124/mol.64.4.876 14500744

[51] Chen X, Geller EB, Rogers TJ, Adler MW (2007). Rapid heterologous desensitization of antinociceptive activity between mu or delta opioid receptors and chemokine receptors in rats. Drug Alcohol Depend.

[52] Grimm MC, Ben-Baruch A, Taub DD, Howard OM, Resau JH, Wang JM, Ali H, Richardson R, Snyderman R, Oppenheim JJ. Opiates transdeactivate chemokine receptors: δ and μ opiate receptor-mediated heterologous desensitization. J Exp Med. 1998;188:317–25. 10.1084/jem.188.2.317 PMC2212445 9670044

[53] Szabo I, Wetzel MA, Zhang N, Steele AD, Kaminsky DE, Chen C, Liu-Chen LY, Bednar F, Henderson EE, Howard OM (2003). Selective inactivation of CCR5 and decreased infectivity of R5 HIV-1 strains mediated by opioid-induced heterologous desensitization. J Leukoc Biol.

[54] Gonek M, McLane VD, Stevens DL, Lippold K, Akbarali HI, Knapp PE, Dewey WL, Hauser KF, Paris JJ (2018). CCR5 mediates HIV-1 Tat-induced neuroinflammation and influences morphine tolerance, dependence, and reward. Brain Behav Immun.

[55] Kleinnijenhuis J, Quintin J, Preijers F, Joosten LA, Ifrim DC, Saeed S, Jacobs C, van Loenhout J, de Jong D, Stunnenberg HG (2012). Bacille Calmette-Guerin induces NOD2-dependent nonspecific protection from reinfection via epigenetic reprogramming of monocytes. Proc Natl Acad Sci U S A.

[56] Shalova IN, Lim JY, Chittezhath M, Zinkernagel AS, Beasley F, Hernandez-Jimenez E, Toledano V, Cubillos-Zapata C, Rapisarda A, Chen J, et al. Human monocytes undergo functional re-programming during sepsis mediated by hypoxia-inducible factor-1α. Immunity. 2015;42:484–98. 10.1016/j.immuni.2015.02.001 25746953

[57] Wendeln AC, Degenhardt K, Kaurani L, Gertig M, Ulas T, Jain G, Wagner J, Hasler LM, Wild K, Skodras A (2018). Innate immune memory in the brain shapes neurological disease hallmarks. Nature.

[58] Nass SR, Hahn YK, McLane VD, Varshneya NB, Damaj MI, Knapp PE, Hauser KF (2020). Chronic HIV-1 Tat exposure alters anterior cingulate cortico-basal ganglia-thalamocortical synaptic circuitry, associated behavioral control, and immune regulation in male mice. Brain, Behavior, and Immunity - Health.

[59] Ning T, Leng C, Chen L, Ma B, Gong X. Metabolomics analysis of serum in a rat heroin self-administration model undergoing reinforcement based on ^1^H-nuclear magnetic resonance spectra. BMC Neurosci. 2018;19:4. 10.1186/s12868-018-0404-5 PMC5836429 29502536

[60] Li RS, Takeda T, Ohshima T, Yamada H, Ishii Y (2017). Metabolomic profiling of brain tissues of mice chronically exposed to heroin. Drug Metab Pharmacokinet.

[61] Ledent C, Valverde O, Cossu G, Petitet F, Aubert JF, Beslot F, Bohme GA, Imperato A, Pedrazzini T, Roques BP, et al. Unresponsiveness to cannabinoids and reduced addictive effects of opiates in CB_1_ receptor knockout mice. Science. 1999;283:401–4. 10.1126/science.283.5400.401 9888857

[62] Navarro M, Carrera MR, Fratta W, Valverde O, Cossu G, Fattore L, Chowen JA, Gomez R, del Arco I, Villanua MA (2001). Functional interaction between opioid and cannabinoid receptors in drug self-administration. J Neurosci.

[63] Yamaguchi T, Hagiwara Y, Tanaka H, Sugiura T, Waku K, Shoyama Y, Watanabe S, Yamamoto T (2001). Endogenous cannabinoid, 2-arachidonoylglycerol, attenuates naloxone-precipitated withdrawal signs in morphine-dependent mice. Brain Res.

[64] Wilkerson JL, Ghosh S, Mustafa M, Abdullah RA, Niphakis MJ, Cabrera R, Maldonado RL, Cravatt BF, Lichtman AH (2017). The endocannabinoid hydrolysis inhibitor SA-57: Intrinsic antinociceptive effects, augmented morphine-induced antinociception, and attenuated heroin seeking behavior in mice. Neuropharmacology.

[65] de Guglielmo G, Kallupi M, Scuppa G, Stopponi S, Demopulos G, Gaitanaris G, Ciccocioppo R. Analgesic tolerance to morphine is regulated by PPARγ. Br J Pharmacol. 2014;171:5407–16. 10.1111/bph.12851 PMC4294048 25048682

[66] Kaczocha M, Azim S, Nicholson J, Rebecchi MJ, Lu Y, Feng T, Romeiser JL, Reinsel R, Rizwan S, Shodhan S (2018). Intrathecal morphine administration reduces postoperative pain and peripheral endocannabinoid levels in total knee arthroplasty patients: a randomized clinical trial. BMC Anesthesiol.

[67] Rubino T, Tizzoni L, Vigano D, Massi P, Parolaro D (1997). Modulation of rat brain cannabinoid receptors after chronic morphine treatment. Neuroreport.

[68] Gonzalez S, Fernandez-Ruiz J, Sparpaglione V, Parolaro D, Ramos JA. Chronic exposure to morphine, cocaine or ethanol in rats produced different effects in brain cannabinoid CB_1_ receptor binding and mRNA levels. Drug Alcohol Depend. 2002;66:77–84. 10.1016/s0376-8716(01)00186-7 11850139

[69] Vigano D, Grazia Cascio M, Rubino T, Fezza F, Vaccani A, Di Marzo V, Parolaro D (2003). Chronic morphine modulates the contents of the endocannabinoid, 2-arachidonoyl glycerol, in rat brain. Neuropsychopharmacology.

[70] Gamage TF, Ignatowska-Jankowska BM, Muldoon PP, Cravatt BF, Damaj MI, Lichtman AH (2015). Differential effects of endocannabinoid catabolic inhibitors on morphine withdrawal in mice. Drug Alcohol Depend.

[71] Wilkerson JL, Niphakis MJ, Grim TW, Mustafa MA, Abdullah RA, Poklis JL, Dewey WL, Akbarali H, Banks ML, Wise LE (2016). The selective monoacylglycerol lipase inhibitor MJN110 produces opioid-sparing effects in a mouse neuropathic pain model. J Pharmacol Exp Ther.

[72] Vigano D, Valenti M, Cascio MG, Di Marzo V, Parolaro D, Rubino T (2004). Changes in endocannabinoid levels in a rat model of behavioural sensitization to morphine. Eur J Neurosci.

[73] Chen D, Liu Y, He W, Wang H, Wang Z (2012). Neurotransmitter-precursor-supplement intervention for detoxified heroin addicts. J Huazhong Univ Sci Technolog Med Sci.

[74] Saxena RN, Pendse VK, Khanna NK (1984). Anti-inflammatory and analgesic properties of four amino-acids. Indian J Physiol Pharmacol.

[75] Liu Y, Wang X, Hu CA (2017). Therapeutic potential of amino acids in inflammatory Bowel disease. Nutrients.

[76] Zhenyukh O, Civantos E, Ruiz-Ortega M, Sanchez MS, Vazquez C, Peiro C, Egido J, Mas S (2017). High concentration of branched-chain amino acids promotes oxidative stress, inflammation and migration of human peripheral blood mononuclear cells via mTORC1 activation. Free Radic Biol Med.

[77] Petrosino S, Palazzo E, de Novellis V, Bisogno T, Rossi F, Maione S, Di Marzo V (2007). Changes in spinal and supraspinal endocannabinoid levels in neuropathic rats. Neuropharmacology.

[78] Mitrirattanakul S, Ramakul N, Guerrero AV, Matsuka Y, Ono T, Iwase H, Mackie K, Faull KF, Spigelman I (2006). Site-specific increases in peripheral cannabinoid receptors and their endogenous ligands in a model of neuropathic pain. Pain.

[79] Hansen HH, Schmid PC, Bittigau P, Lastres-Becker I, Berrendero F, Manzanares J, Ikonomidou C, Schmid HH, Fernandez-Ruiz JJ, Hansen HS (2001). Anandamide, but not 2-arachidonoylglycerol, accumulates during in vivo neurodegeneration. J Neurochem.

[80] Panikashvili D, Simeonidou C, Ben-Shabat S, Hanus L, Breuer A, Mechoulam R, Shohami E (2001). An endogenous cannabinoid (2-AG) is neuroprotective after brain injury. Nature.

[81] Tanveer R, Macguinness N, Daniel S, Gowran A, Campell VA (2012). Cannabinoid receptors and neurodegenerative diseases. WIREs Membr Transp Signal.

[82] Fookes CJ, Pham TQ, Mattner F, Greguric I, Loc'h C, Liu X, Berghofer P, Shepherd R, Gregoire MC, Katsifis A. Synthesis and biological evaluation of substituted [^18^F]imidazo[1,2-*a*]pyridines and [^18^F]pyrazolo[1,5-*a*]pyrimidines for the study of the peripheral benzodiazepine receptor using positron emission tomography. J Med Chem. 2008;51:3700–12. 10.1021/jm7014556 18557607

[83] Van Camp N, Boisgard R, Kuhnast B, Theze B, Viel T, Gregoire MC, Chauveau F, Boutin H, Katsifis A, Dolle F, Tavitian B. In vivo imaging of neuroinflammation: a comparative study between [^18^F]PBR111, [^11^C]CLINME and [^11^C]PK11195 in an acute rodent model. Eur J Nucl Med Mol Imaging. 2010;37:962–72. 10.1007/s00259-009-1353-0 20069292

[84] Callaghan PD, Wimberley CA, Rahardjo GL, Berghofer PJ, Pham TQ, Jackson T, Zahra D, Bourdier T, Wyatt N, Greguric I, et al. Comparison of in vivo binding properties of the 18-kDa translocator protein (TSPO) ligands [^18^F]PBR102 and [^18^F]PBR111 in a model of excitotoxin-induced neuroinflammation. Eur J Nucl Med Mol Imaging. 2015;42:138–51. 10.1007/s00259-014-2895-3 25231248

[85] Hahn YK, Podhaizer EM, Farris SP, Miles MF, Hauser KF, Knapp PE (2013). Effects of chronic HIV-1 Tat exposure in the CNS: heightened vulnerability of males versus females to changes in cell numbers, synaptic integrity, and behavior. Brain Struct Funct.

[86] Bryant CD, Eitan S, Sinchak K, Fanselow MS, Evans CJ (2006). NMDA receptor antagonism disrupts the development of morphine analgesic tolerance in male, but not female C57BL/6J mice. Am J Physiol Regul Integr Comp Physiol.

[87] Bryant CD, Roberts KW, Byun JS, Fanselow MS, Evans CJ (2006). Morphine analgesic tolerance in 129P3/J and 129S6/SvEv mice. Pharmacol Biochem Behav.

[88] Harris LS, Pierson AK (1964). Some narcotic antagonists in the benzomorphan series. J Pharmacol Exp Ther.

[89] Dollé F, Hinnen F, Damont A, Kuhnast B, Fookes C, Pham T, Tavitian B, Katsifis A. Radiosynthesis of [^18^F]PBR111, a selective radioligand for imaging the translocator protein (18 kDa) with PET. Journal of Labelled Compounds and Radiopharmaceuticals. 2008;51:435–9.

[90] Besnard J, Ruda GF, Setola V, Abecassis K, Rodriguiz RM, Huang XP, Norval S, Sassano MF, Shin AI, Webster LA (2012). Automated design of ligands to polypharmacological profiles. Nature.

[91] Kuhnast B, Damont A, Hinnen F, Catarina T, Demphel S, Le Helleix S, Coulon C, Goutal S, Gervais P. Dolle F: [^18^F]DPA-714, [^18^F]PBR111 and [^18^F]FEDAA1106-selective radioligands for imaging TSPO 18 kDa with PET: automated radiosynthesis on a TRACERLAb FX-FN synthesizer and quality controls. Appl Radiat Isot. 2012;70:489–97. 10.1016/j.apradiso.2011.10.015 22104496

[92] McGill BE, Barve RA, Maloney SE, Strickland A, Rensing N, Wang PL, Wong M, Head R, Wozniak DF, Milbrandt J. Abnormal microglia and enhanced inflammation-related gene transcription in mice with conditional deletion of *Ctcf* in *Camk2a*-Cre-expressing neurons. J Neurosci. 2018;38:200–19. 10.1523/JNEUROSCI.0936-17.2017 PMC5761433 29133437

[93] Young K, Morrison H (2018). Quantifying microglia morphology from photomicrographs of immunohistochemistry prepared tissue using imagej. J Vis Exp.

[94] Fitting S, Scoggins KL, Xu R, Dever SM, Knapp PE, Dewey WL, Hauser KF (2012). Morphine efficacy is altered in conditional HIV-1 Tat transgenic mice. Eur J Pharmacol.

[95] Gouveia-Figueira S, Nording ML (2015). Validation of a tandem mass spectrometry method using combined extraction of 37 oxylipins and 14 endocannabinoid-related compounds including prostamides from biological matrices. Prostaglandins Other Lipid Mediat.

[96] Preinerstorfer B, Schiesel S, Lammerhofer M, Lindner W (2010). Metabolic profiling of intracellular metabolites in fermentation broths from beta-lactam antibiotics production by liquid chromatography-tandem mass spectrometry methods. J Chromatogr A.

[97] Greenhouse SW, Geisser S (1959). On methods in the analysis of profile data. Psychometrika.

[98] Fitting S, Stevens DL, Khan FA, Scoggins KL, Enga RM, Beardsley PM, Knapp PE, Dewey WL, Hauser KF (2016). Morphine tolerance and physical dependence are altered in conditional HIV-1 Tat transgenic mice. J Pharmacol Exp Ther.

[99] Casellas P, Galiegue S, Basile AS (2002). Peripheral benzodiazepine receptors and mitochondrial function. Neurochem Int.

[100] Braestrup C, Squires RF. Specific benzodiazepine receptors in rat brain characterized by high-affinity [^3^H]diazepam binding. Proc Natl Acad Sci U S A. 1977;74:3805–9. 10.1073/pnas.74.9.3805 PMC431738 20632

[101] Duncan MJ, Bruce-Keller AJ, Conner C, Knapp PE, Xu R, Nath A, Hauser KF (2008). Effects of chronic expression of the HIV-induced protein, transactivator of transcription, on circadian activity rhythms in mice, with or without morphine. Am J Physiol Regul Integr Comp Physiol.

[102] Suryawan A, Hawes JW, Harris RA, Shimomura Y, Jenkins AE, Hutson SM (1998). A molecular model of human branched-chain amino acid metabolism. Am J Clin Nutr.

[103] Fernstrom JD (1990). Aromatic amino acids and monoamine synthesis in the central nervous system: influence of the diet. J Nutr Biochem.

[104] Daikhin Y, Yudkoff M (2000). Compartmentation of brain glutamate metabolism in neurons and glia. J Nutr.

[105] Lee JH, Park E, Jin HJ, Lee Y, Choi SJ, Lee GW, Chang PS, Paik HD (2017). Anti-inflammatory and anti-genotoxic activity of branched chain amino acids (BCAA) in lipopolysaccharide (LPS) stimulated RAW 264.7 macrophages. Food Sci Biotechnol.

[106] Fernstrom JD (2005). Branched-chain amino acids and brain function. J Nutr.

[107] Pocernich CB, Sultana R, Mohmmad-Abdul H, Nath A, Butterfield DA (2005). HIV-dementia, Tat-induced oxidative stress, and antioxidant therapeutic considerations. Brain Res Brain Res Rev.

[108] Kim SH, Smith AJ, Tan J, Shytle RD, Giunta B (2015). MSM ameliorates HIV-1 Tat induced neuronal oxidative stress via rebalance of the glutathione cycle. Am J Transl Res.

[109] El-Amine R, Germini D, Zakharova VV, Tsfasman T, Sheval EV, Louzada RAN, Dupuy C, Bilhou-Nabera C, Hamade A, Najjar F (2018). HIV-1 Tat protein induces DNA damage in human peripheral blood B-lymphocytes via mitochondrial ROS production. Redox Biol.

[110] Fernstrom JD, Fernstrom MH (2007). Tyrosine, phenylalanine, and catecholamine synthesis and function in the brain. J Nutr.

[111] Nolan R, Gaskill PJ (1702). The role of catecholamines in HIV neuropathogenesis. Brain Res.

[112] Gostner JM, Becker K, Kurz K, Fuchs D (2015). Disturbed amino acid metabolism in HIV: association with neuropsychiatric symptoms. Front Psychiatry.

[113] Lee J, Lee JY, Meade CS, Cohn M, Chahine A, Dilworth SE, Magidson JF, Gouse H, Fuchs D, Carrico AW: Tryptophan degradation is associated with risk-taking propensity in methamphetamine users with treated HIV infection. J Neurovirol. 2020. Online ahead of print. 10.1007/s13365-020-00841-4. 10.1007/s13365-020-00841-4 PMC7541781 32728896

[114] Kumar AM, Ownby RL, Waldrop-Valverde D, Fernandez B, Kumar M (2011). Human immunodeficiency virus infection in the CNS and decreased dopamine availability: relationship with neuropsychological performance. J Neurovirol.

[115] Nath A, Anderson C, Jones M, Maragos W, Booze R, Mactutus C, Bell J, Hauser KF, Mattson M (2000). Neurotoxicity and dysfunction of dopaminergic systems associated with AIDS dementia. J Psychopharmacol.

[116] Cass WA, Harned ME, Peters LE, Nath A, Maragos WF (2003). HIV-1 protein Tat potentiation of methamphetamine-induced decrease in evoked overflow of dopamine in the striatum of the rat. Brain Research.

[117] Gaskill PJ, Miller DR, Gamble-George J, Yano H, Khoshbouei H (2017). HIV, Tat and dopamine transmission. Neurobiol Dis.

[118] Sun WL, Quizon PM, Yuan Y, Strauss MJ, McCain R, Zhan CG, Zhu J (2019). Mutational effects of human dopamine transporter at tyrosine88, lysine92, and histidine547 on basal and HIV-1 Tat-inhibited dopamine transport. Sci Rep.

[119] Andrade VS, Rojas DB, de Andrade RB, Kim TDH, Vizuete AF, Zanatta A, Wajner M, Goncalves CS, Wannmacher CMD (2018). A possible anti-inflammatory effect of proline in the brain cortex and cerebellum of rats. Mol Neurobiol.

[120] Perrot S, Guilbaud G, Kayser V (1999). Effects of intraplantar morphine on paw edema and pain-related behaviour in a rat model of repeated acute inflammation. Pain.

[121] Rodriguez AE, Ducker GS, Billingham LK, Martinez CA, Mainolfi N, Suri V, Friedman A, Manfredi MG, Weinberg SE, Rabinowitz JD, Chandel NS (2019). Serine metabolism supports macrophage IL-1beta production. Cell Metab.

[122] Zhai PP, Xu LH, Yang JJ, Jiang ZL, Zhao GW, Sun L, Wang GH, Li X (2015). Reduction of inflammatory responses by L-serine treatment leads to neuroprotection in mice after traumatic brain injury. Neuropharmacology.

[123] Robin LM, Oliveira da Cruz JF, Langlais VC, Martin-Fernandez M, Metna-Laurent M, Busquets-Garcia A, Bellocchio L, Soria-Gomez E, Papouin T, Varilh M, et al. Astroglial CB_1_ receptors determine synaptic D-serine availability to enable recognition memory. Neuron. 2018;98:935–44 e935. 10.1016/j.neuron.2018.04.034 29779943

[124] Borrelli F, Izzo AA (2009). Role of acylethanolamides in the gastrointestinal tract with special reference to food intake and energy balance. Best Pract Res Clin Endocrinol Metab.

[125] Borrelli F, Romano B, Petrosino S, Pagano E, Capasso R, Coppola D, Battista G, Orlando P, Di Marzo V, Izzo AA (2015). Palmitoylethanolamide, a naturally occurring lipid, is an orally effective intestinal anti-inflammatory agent. Br J Pharmacol.

[126] Fu J, Gaetani S, Oveisi F, Lo Verme J, Serrano A, Rodriguez De Fonseca F, Rosengarth A, Luecke H, Di Giacomo B, Tarzia G, Piomelli D (2003). Oleylethanolamide regulates feeding and body weight through activation of the nuclear receptor PPAR-alpha. Nature.

[127] Lo Verme J, Gaetani S, Fu J, Oveisi F, Burton K, Piomelli D (2005). Regulation of food intake by oleoylethanolamide. Cell Mol Life Sci.

[128] Ahern GP (2003). Activation of TRPV1 by the satiety factor oleoylethanolamide. J Biol Chem.

[129] Suardiaz M, Estivill-Torrus G, Goicoechea C, Bilbao A, Rodriguez de Fonseca F (2007). Analgesic properties of oleoylethanolamide (OEA) in visceral and inflammatory pain. Pain.

[130] Wang X, Miyares RL, Ahern GP (2005). Oleoylethanolamide excites vagal sensory neurones, induces visceral pain and reduces short-term food intake in mice via capsaicin receptor TRPV1. J Physiol.

[131] Artmann A, Petersen G, Hellgren LI, Boberg J, Skonberg C, Nellemann C, Hansen SH, Hansen HS (1781). Influence of dietary fatty acids on endocannabinoid and N-acylethanolamine levels in rat brain, liver and small intestine. Biochim Biophys Acta.

[132] Nomura DK, Blankman JL, Simon GM, Fujioka K, Issa RS, Ward AM, Cravatt BF, Casida JE (2008). Activation of the endocannabinoid system by organophosphorus nerve agents. Nat Chem Biol.

[133] Bisogno T, Martire A, Petrosino S, Popoli P, Di Marzo V (2008). Symptom-related changes of endocannabinoid and palmitoylethanolamide levels in brain areas of R6/2 mice, a transgenic model of Huntington's disease. Neurochem Int.

[134] Loria F, Petrosino S, Mestre L, Spagnolo A, Correa F, Hernangomez M, Guaza C, Di Marzo V, Docagne F (2008). Study of the regulation of the endocannabinoid system in a virus model of multiple sclerosis reveals a therapeutic effect of palmitoylethanolamide. Eur J Neurosci.

[135] Rubio M, McHugh D, Fernandez-Ruiz J, Bradshaw H, Walker JM (2007). Short-term exposure to alcohol in rats affects brain levels of anandamide, other N-acylethanolamines and 2-arachidonoyl-glycerol. Neurosci Lett.

[136] Schabitz WR, Giuffrida A, Berger C, Aschoff A, Schwaninger M, Schwab S, Piomelli D (2002). Release of fatty acid amides in a patient with hemispheric stroke: a microdialysis study. Stroke.

[137] Lo Verme J, Fu J, Astarita G, La Rana G, Russo R, Calignano A, Piomelli D. The nuclear receptor peroxisome proliferator-activated receptor-α mediates the anti-inflammatory actions of palmitoylethanolamide. Mol Pharmacol. 2005;67:15–9. 10.1124/mol.104.006353 15465922

[138] Guzman M, Lo Verme J, Fu J, Oveisi F, Blazquez C, Piomelli D. Oleoylethanolamide stimulates lipolysis by activating the nuclear receptor peroxisome proliferator-activated receptor α (PPAR-α). J Biol Chem. 2004;279:27849–54. 10.1074/jbc.M404087200 15123613

[139] Hansen HS (2010). Palmitoylethanolamide and other anandamide congeners. Proposed role in the diseased brain. Exp Neurol.

[140] Maccarrone M, Cartoni A, Parolaro D, Margonelli A, Massi P, Bari M, Battista N, Finazzi-Agro A (2002). Cannabimimetic activity, binding, and degradation of stearoylethanolamide within the mouse central nervous system. Mol Cell Neurosci.

[141] Hong S, Banks WA (2015). Role of the immune system in HIV-associated neuroinflammation and neurocognitive implications. Brain Behav Immun.

[142] Zou W, Kim BO, Zhou BY, Liu Y, Messing A, He JJ. Protection against human immunodeficiency virus type 1 Tat neurotoxicity by *Ginkgo biloba* extract EGb 761 involving glial fibrillary acidic protein. Am J Pathol. 2007;171:1923–35. 10.2353/ajpath.2007.070333 PMC2111115 18055541

[143] Boska MD, Dash PK, Knibbe J, Epstein AA, Akhter SP, Fields N, High R, Makarov E, Bonasera S, Gelbard HA (2014). Associations between brain microstructures, metabolites, and cognitive deficits during chronic HIV-1 infection of humanized mice. Mol Neurodegener.

[144] Amhaoul H, Hamaide J, Bertoglio D, Reichel SN, Verhaeghe J, Geerts E, Van Dam D, De Deyn PP, Kumar-Singh S, Katsifis A (2015). Brain inflammation in a chronic epilepsy model: Evolving pattern of the translocator protein during epileptogenesis. Neurobiol Dis.

[145] Dedeurwaerdere S, Callaghan PD, Pham T, Rahardjo GL, Amhaoul H, Berghofer P, Quinlivan M, Mattner F, Loc'h C, Katsifis A, Gregoire MC (2012). PET imaging of brain inflammation during early epileptogenesis in a rat model of temporal lobe epilepsy. EJNMMI Res.

[146] Mattner F, Staykova M, Berghofer P, Wong HJ, Fordham S, Callaghan P, Jackson T, Pham T, Gregoire MC, Zahra D (2013). Central nervous system expression and PET imaging of the translocator protein in relapsing-remitting experimental autoimmune encephalomyelitis. J Nucl Med.

[147] Kosten L, Verhaeghe J, Verkerk R, Thomae D, De Picker L, Wyffels L, Van Eetveldt A, Dedeurwaerdere S, Stroobants S, Staelens S (2016). Multiprobe molecular imaging of an NMDA receptor hypofunction rat model for glutamatergic dysfunction. Psychiatry Res Neuroimaging.

[148] Jaeger LB, Nath A (2012). Modeling HIV-associated neurocognitive disorders in mice: new approaches in the changing face of HIV neuropathogenesis. Dis Model Mech.

[149] Hauser KF, Hahn YK, Adjan VV, Zou S, Buch SK, Nath A, Bruce-Keller AJ, Knapp PE (2009). HIV-1 Tat and morphine have interactive effects on oligodendrocyte survival and morphology. Glia.

[150] Pannell M, Economopoulos V, Wilson TC, Kersemans V, Isenegger PG, Larkin JR, Smart S, Gilchrist S, Gouverneur V, Sibson NR (2019). Imaging of translocator protein upregulation is selective for pro-inflammatory polarized astrocytes and microglia. Glia.

[151] Beckers L, Ory D, Geric I, Declercq L, Koole M, Kassiou M, Bormans G, Baes M (2018). Increased expression of translocator protein (TSPO) marks pro-inflammatory microglia but does not predict neurodegeneration. Mol Imaging Biol.

[152] Betlazar C, Middleton RJ, Banati R, Liu GJ (2020). The Translocator Protein (TSPO) in Mitochondrial Bioenergetics and Immune Processes. Cells.

[153] Ransohoff RM (2016). A polarizing question: do M1 and M2 microglia exist?. Nat Neurosci.

[154] Coughlin JM, Wang Y, Ma S, Yue C, Kim PK, Adams AV, Roosa HV, Gage KL, Stathis M, Rais R, et al. Regional brain distribution of translocator protein using [^11^C]DPA-713 PET in individuals infected with HIV. J Neurovirol. 2014;20:219–32. 10.1007/s13365-014-0239-5 PMC4078660 24567030

[155] Chen MK, Guilarte TR (2008). Translocator protein 18 kDa (TSPO): molecular sensor of brain injury and repair. Pharmacol Ther.

[156] Ji B, Maeda J, Sawada M, Ono M, Okauchi T, Inaji M, Zhang MR, Suzuki K, Ando K, Staufenbiel M (2008). Imaging of peripheral benzodiazepine receptor expression as biomarkers of detrimental versus beneficial glial responses in mouse models of Alzheimer's and other CNS pathologies. J Neurosci.

[157] Cosenza-Nashat M, Zhao ML, Suh HS, Morgan J, Natividad R, Morgello S, Lee SC (2009). Expression of the translocator protein of 18 kDa by microglia, macrophages and astrocytes based on immunohistochemical localization in abnormal human brain. Neuropathol Appl Neurobiol.

[158] Chen MK, Guilarte TR (2006). Imaging the peripheral benzodiazepine receptor response in central nervous system demyelination and remyelination. Toxicol Sci.

[159] Lajqi T, Lang GP, Haas F, Williams DL, Hudalla H, Bauer M, Groth M, Wetzker R, Bauer R (2019). Memory-like inflammatory responses of microglia to rising doses of LPS: key role of PI3Kgamma. Front Immunol.

[160] Schaafsma W, Zhang X, van Zomeren KC, Jacobs S, Georgieva PB, Wolf SA, Kettenmann H, Janova H, Saiepour N, Hanisch UK (2015). Long-lasting pro-inflammatory suppression of microglia by LPS-preconditioning is mediated by RelB-dependent epigenetic silencing. Brain Behav Immun.

[161] Holtman IR, Raj DD, Miller JA, Schaafsma W, Yin Z, Brouwer N, Wes PD, Moller T, Orre M, Kamphuis W (2015). Induction of a common microglia gene expression signature by aging and neurodegenerative conditions: a co-expression meta-analysis. Acta Neuropathol Commun.

[162] Lederman MM, Funderburg NT, Sekaly RP, Klatt NR, Hunt PW (2013). Residual immune dysregulation syndrome in treated HIV infection. Adv Immunol.

[163] Fischer-Smith T, Rappaport J (2005). Evolving paradigms in the pathogenesis of HIV-1-associated dementia. Expert Rev Mol Med.

[164] Spudich SS (2016). Immune activation in the central nervous system throughout the course of HIV infection. Curr Opin HIV AIDS.

[165] Swanta N, Aryal S, Nejtek V, Shenoy S, Ghorpade A, Borgmann K (2020). Blood-based inflammation biomarkers of neurocognitive impairment in people living with HIV. J Neurovirol.

[166] Chen K, Phan T, Lin A, Sardo L, Mele AR, Nonnemacher MR, Klase Z (2020). Morphine exposure exacerbates HIV-1 Tat driven changes to neuroinflammatory factors in cultured astrocytes. PLoS One.

